# KCTD15 Is Overexpressed in her2+ Positive Breast Cancer Patients and Its Silencing Attenuates Proliferation in SKBR3 CELL LINE

**DOI:** 10.3390/diagnostics12030591

**Published:** 2022-02-25

**Authors:** Luigi Coppola, Simona Baselice, Francesco Messina, Rosa Giannatiempo, Amalia Farina, Luigi Vitagliano, Giovanni Smaldone, Marco Salvatore

**Affiliations:** 1 IRCCS SYNLAB SDN S.p.a., Napoli, Via E. Gianturco 113, 80143 Napoli, Italy; luigi.coppola@synlab.it (L.C.); simona.baselice@synlab.it (S.B.); direzionescientifica.irccssdn@synlab.it (M.S.); 2 Ospedale Evangelico Betania, Via Argine 604, 80147 Napoli, Italy; messina52@alice.it (F.M.); giannatiemporosa@libero.it (R.G.); amaliafarina@hotmail.it (A.F.); 3 Institute of Biostructures and Bioimaging, C.N.R., 80134 Napoli, Italy

**Keywords:** breast cancer, KCTD15, HER2+, breast cancer subtypes, biomarker

## Abstract

Studies carried out in the last decade have demonstrated that the members of the KCTD protein family play active roles in carcinogenesis. Very recently, it has been reported that KCTD15, a protein typically associated with other physio-pathological processes, is involved in medulloblastoma and leukemia. Starting with some preliminary indications that emerged from the analysis of online databases that suggested a possible overexpression of KCTD15 in breast cancer, in this study, we evaluated the expression levels of the protein in breast cancer cell lines and in patients and the effects of its silencing in the HER2+ cell model. The analysis of the KCTD15 levels indicates a significant overexpression of the protein in Luminal A and Luminal B breast cancer patients as well as in the related cell lines. The greatest level of over-expression of the protein was found in HER2+ patients and in the related SKBR3 cell line model system. The effects of KCTD15 silencing in terms of cell proliferation, cell cycle, and sensitivity to doxorubicin were evaluated in the SKBR3 cell line. Notably, the KCTD15 silencing in SKBR3 cells by CRISPR/CAS9 technology significantly attenuates their proliferation and cell cycle progression. Finally, we demonstrated that KCT15 silencing also sensitized SKBR3 cells to the cytotoxic agent doxorubicin, suggesting a possible role of the protein in anti HER2+ therapeutic strategies. Our results highlight a new possible player in HER2 breast cancer carcinogenesis, paving the way for its use in breast cancer diagnosis and therapy.

## 1. Introduction

Studies carried out in the last decade have clearly shown that the members of the protein family, known as KCTD (Potassium(K) Channel Tetramerization Domain containing proteins) are involved in diversified yet fundamental biological processes [1,2,3,4,5]. There is clear and convergent evidence that these proteins play important roles in neurodevelopmental and neuropsychiatric disorders [2]. These include schizophrenia and autism (KCTD13), neurocognitive disorders (KCTD3), epilepsy (KCTD7), and movement disorders (KCTD17). However, surveys of literature data indicate that they are also involved in obesity, genetic diseases, and cancer [5,6,7,8]. In oncology, these proteins have been associated with medulloblastoma, breast cancer (BC), prostate adenocarcinoma, osteosarcoma, lung cancer, neuroendocrine tumors, and gastrointestinal stromal tumor [8,9,10]. These diversified roles may be, in principle, ascribed to the distinctive molecular properties exhibited by different members of the family. Indeed, while sharing a BTB/POZ domain at the N-terminus of their sequences, the C-terminal regions of the KCTD proteins present highly diversified and frequently unrelated sequences. Similarities at the C-terminus are evident only for KCTD members belonging to the same clade of the phylogenetic tree of the family. Intriguingly, Specific members of the family or members of the same clade also have the ability to play roles in distinct physio-pathological processes. A remarkable example of this situation is represented by the proteins KCTD1 and KCTD15 that belong to clade A and that share a remarkable sequence identity (~80%). Both proteins can inhibit neural-crest formation by attenuating the WNT/β-catenin pathway [11,12] and are involved in adipogenesis and/or obesity [6,13,14,15]. Natural mutants of KCTD1 have been demonstrated to be the causative agents of the scalp-ear-nipple syndrome [7,16] whereas KCTD15 inhibits the Hedgehog pathway in medulloblastoma cells [8]. We have recently shown that KCTD15 is overexpressed in patients and cell lines of both childhood B-cell acute lymphoid leukemia and acute myeloid leukemia [17,18]. We have also shown that KCTD15 also displays a characteristic and well-defined profile of expression in peripheral blood cells (granulocytes, monocytes, and lymphocytes) [17]. Although the biochemical role(s) underlying this assortment of biological functions are yet to be uncovered, the observation that KCTD15 silencing-induced apoptosis and cell death in leukemia cell suggesting that it has a role in cellular homeostasis and proliferation prompted us an analysis of the KCTD15 expression levels in other oncological conditions. In this scenario, we have recently shown that KCTD15 overexpression is associated with an activation of the NF-κB pathway, which likely occurs through an upregulation of IKK phosphorylation [19]. A connection between the KCTD15 and the NF-κB pathway has also been detected in the breast cancer cell line SKBR3 [19].

Here, we present an analysis of the expression level of KCTD15 in different BC tissues subtypes as well as in different human cell lines. Breast cancer is a highly heterogeneous disease characterized by multiple tumoral subtypes with different biological features whose identification has allowed for a more precise management of patients [20]. The increased understanding of the tumorigenesis process related to breast cancer, and the introduction of innovative technologies, have led to the identification of different subtypes [21,22]. BC can be classified into Luminal A (Estrogen Receptor (ER)+ or Progesterone Receptor (PR)+, and Human Epidermal growth factor Receptor 2 (HER2)−), Luminal B (ER+ or PR+, HER2+), HER2-positve (ER− and PR−, HER2+), and Triple Negative (ER− and PR−, HER2−) subtypes. Although an improved understanding of the tumorigenesis processes related to BC has been achieved in recent decades, the identification of new active players in the etiology of the disease represents a goal with significant therapeutic and diagnostic implications. In this study, we initially quantified KCTD15 expression in BC tissues subtypes as well as in different human cell lines. Moreover, we evaluated KCTD15 silencing in terms of cell proliferation, cell cycle and sensitivity to doxorubicin. Collectively, the data presented suggest that KCTD15 play an active role in the insurgence of specific cases of breast cancer and open new opportunities in the diagnosis and therapy of this this highly heterogeneous disease.

## 2. Materials and Methods

### 2.1. Cell Lines

The non-tumorigenic epithelial MCF10-a cell line and human breast cancer cell lines (SKBR3, MCF7, MDA-MB-231, BT-474, MDA-MB-361, MDA-MB-453 and T47D) were obtained from the American Type Culture Collection. The SKBR3 cells were grown in McCoy’s 5A Medium Modified supplemented with 10% fetal bovine serum (FBS). On the other hand, MCF-7, MDA-MB-231, BT474, MDA-MB-361, and T47D cells were grown in RPMI-1640 medium with GlutaMAX 1% supplemented with 10% FBS, indeed MDA-MB-453 were growth in L-15 medium supplemented with 10% FBS. Finally, MCF10a cells were grown in the MEBM medium (CC-3151, Lonza, Belgium). For the generation of KCTD15 silenced SKBR3 cells, we used the CRISPR/CAS9 technology [23]. Briefly, SKBR3 cells were transfected with KCTD15 double nickase plasmid (sc-407663-NIC-2, Santa Cruz Biotechnology, Inc., Dallas, TX, USA) to obtain KCTD15 silenced clones (SKBR3^KCTD15−^). In addition to the obtained SKBR3 control cells (SKBR3^ctrl^), SKBR3 cells were transfected with CRISPR/CAS9 control plasmid (sc-418922, Santa Cruz Biotechnology, Inc., Dallas, TX, USA) plus pReceiver-M94 plasmid (GeneCopoeia, Inc., Rockville, MD, USA). Transfections were conducted using Lipofectamine 3000 reagent (L3000001, Thermo Fisher Scientific, Waltham, MA, USA) following the manufacturer instructions. Clones were selected with 1.5 µg/mL puromycin. For chemotherapy cytotoxicity assays, both SKBR3^ctrl^ and SKBR3^KCTD15−^ cells were treated with doxorubicin 2 and 5 µM for 16 h. All cell lines were cultured at 37 °C in a humidified atmosphere with 5% CO_2_. Additionally, MDA-MB-453 was used as an incubator without CO_2_.

### 2.2. Online Data and Statistical Analyses

The KCTD15 mRNA expression in normal and tumor tissues was gathered from GEPIA2 (Gene Expression Profiling Interactive Analysis, RRID:SCR_018294), an online platform with RNA-seq data from TCGA and GTEx databases [24,25]. The median values of KCTD15 transcripts, derived either from normal or tumor tissues, were plotted using the one-way analysis of variance (ANOVA) differential method and a q value cutoff of 0.01. Survival curves were generated using two online datasets. The first dataset was the Kaplan–Meier Plotter (KMplotter), an online platform combining gene microarray data and patient survival rates from Gene Expression Omnibus [24]. The second dataset was GEPIA2, an online tool that uses the TCGA breast cancer data [25] (http://gepia.cancer-pku.cn/, last viewed date: 23 November 2020). Patients were divided using an auto-selection feature based on median and quartile expression levels of KCTD15. Median survival was reported in months and compared for significance with a hazard ratio and *p*-value generated on the graph. A *p*-value of <0.05 was considered statistically significant.

### 2.3. RNA Extraction and qRT-PCR

Total RNA was extracted from cultured cells using the Trizol Reagent protocol (Thermo Fischer Scientific, Waltham, MA, USA). After extraction, RNA was quantified with Qubit 4 Fluorometer (Thermo Fischer Scientific). Next, 0.5 µg of total RNA from each sample was reverted in cDNA using SuperScript III First-Strand Synthesis SuperMix kit (Thermo Fisher Scientific) according to the manufacturer’s protocol. The expression level of Cyclins was measured by qRT-PCR using the following formula: 2-∆Ct on C1000 Touch Thermal Cycler (Bio-Rad, Hercules, CA, USA) using iQ SYBR Green Supermix (#1708882, Bio-Rad). Ribosomal Protein S18 (RPS18) level was used as an endogenous control to normalize Cyclins expression.

The following forward (fw) and reverse (rev) primers were used:

- RPS18:
  fw 5′-CGATGGGCGGCGGAAAATA-3′;
  rev 5′-CTGCTTTCCTCAACACCACA-3′
- CyclinA:
  fw 5′-AAATGGGCAGTACAGGAGGA-3′;
  rev 5′-CCACAGTCAGGGAGTGCTTT-3′
- CyclinB:
  fw 5′-CATGGTGCACTTTCCTCCTT-3′;
  rev 5′ AGGTAATGTTGTAGAGTTGGTGTCC-3′
- CyclinE:
  fw 5′-GGCCAAAATCGACAGGAC-3′;
  rev 5′-GGGTCTGCACAGACTGCAT-3′
- CyclinD:
  fw 5′-GCTGTGCATCTACACCGACA-3′;
  rev 5′-TTGAGCTTGTTCACCAGGAG-3′

### 2.4. Western Blot Analysis

Lysates from breast cancer cell lines (50 µg of protein extracts) were analyzed by Western blot to check the levels of protein expression. The following antibodies were used: anti KCTD15 (GTX50002, GeneTex Cat# GTX50002), anti-p27 Kip1 (D69C12, Cell Signaling Technology Cat# 13715), anti-p21 Waf1/Cip1 (Cell Signaling Technology Cat# 2947), anti β-actin (Abcam Cat# ab11004) and anti β-tubulin (Sigma-Aldrich Cat# T0198 as internal controls). Proteins were acquired using the ChemiDoc Imaging System (Bio-Rad, Hercules, CA, USA) coupled with Image Lab software (Bio-Rad, Hercules, CA, USA).

### 2.5. MTT Assay

Cell viability was assessed by the MTT (3-(4,5-dimethylthiazol-2-yl)-2,5-diphenyltetrazolium bromide) assays (G4000, Promega, Italy). Both SKBR3^ctrl^ and SKBR3^KCTD15−^ cells were seeded in 96-well plates at a density of 8 × 10^3^ per well. For drug treatments, cells were incubated with 2 and 5 μM of Doxorubicin for 16h and 40 μg/mL Trastuzumab (HY-P9907, MedChem Express) for 5 days as previously reported [26]. Absorbance values were determined at 490 nm using an automatic plate reader (Victor Nivo, Perkin Elmer, Waltham, MA, USA). MTT assays were repeated thrice with similar results.

### 2.6. Migration Assay

For the migration assay, SKBR3^ctrl^ and SKBR3^KCTD15−^ cells (1 × 10^4^ cells) were resuspended in McCoy medium without FBS in a hollow plastic chamber sealed at one end with a porous membrane and placed in a 24-well plate containing 500 μL of complete McCoy medium (10% FBS). The day after, migrated cells were fixed in frozen methanol, washed with PBS, and stained with blue-fluorescent 4′,6-diamidino-2-phenylindole dihydrochloride (DAPI, Sigma-Aldrich, St. Louis, MO, USA) and Crystal violet following the manufacture protocols. Dapi labelled cells were acquired using a microscope (Leica DM5500 B) coupled with Leica Cytovision software, while Crystal violet was quantified using an automatic plate reader (Victor Nivo, Perkin Elmer, 590 nm).

### 2.7. Flow Cytometry Experiments

Furthermore, Cytoflex V2-B4-R2 (Beckman-Coulter, Brea, CA, USA) was used to evaluate the expression of the KCTD15 protein in breast cancer cells. QC Fluorospheres were used preliminary before each experiment to verify the flow cytometer’s optical alignment and fluidics system. Intracellular or combined intracellular plus surface staining was performed using the PerFix Expose kit (B26976, Beckman Coulter, Brea, CA, USA) according to manufacturer instruction. Briefly, it consists of three ready-to-use reagents and a final solution requiring a 20-fold dilution before use. This kit is used to prepare biological samples for the analysis of intracellular determinants by Flow Cytometry (FCM). Additionally, it can detect several surface antigens together with intracellular markers. In the present study, the PerFix Expose kit was used for the detection of KCTD15 intracellular expression in the SKBR3, T47D, MDA-MB453, MDA-MB-361, MDA-MB-231, MCF-7, BT-474, and MCF-10A cells. These cells were fixed and permeabilized using Buffer 1 and 2 according to the manufacturer instructions. Then, antibody staining was performed for 30 min in buffer 3 reagent using unconjugated anti KCTD15 monoclonal antibody (GeneTex Cat# GTX50002, Irvine, CA, USA).

For all of the analyzed cell lines, we subtracted the background of the isotypic control. For Cell Cycle analysis, a minimum of 10,000 single cell events were recorded using DNA-PREP REAGENTS KIT (6607055, Beckman Coulter). Cell cycle analysis was performed using Kaluza Analysis Software 2.1 (Beckman Coulter) with the Michael Fox algorithm to determine G1, S and G2/M phases [27].

### 2.8. Microscopy Analyses

For immunofluorescence evaluation on cancer cells, SKBR3 cells (3 × 10^4^/well) were plated in 6-well plates that were previously fitted with four cylindrical glasses at the base. After 24 h, cells were fixed with a 1:1 solution of methanol/acetone for 10 min at a temperature of −80 °C and then incubated with 1% bovine serum albumin in DPBS for 1 h. Cells were firstly incubated with the primary antibody Actin, (1:50) for 2 h at room temperature (Sigma-Aldrich Cat# A2228), and afterwards with a FITC labeled secondary mouse antibody for 1 h at room temperature (Abcam Cat# ab7064). The KCTD15 primary antibody (1:50) (GeneTex Cat# GTX50002) was incubated simultaneously. A PE-labelled secondary rabbit antibody (Sigma-Aldrich Cat# P9537) was used for the detection of KCTD15. Nuclei were stained in blue with DAPI (1:30,000) for 10 min at room temperature (Thermo Fisher Scientific Cat# D1306). For immunofluorescence and phase-contrast images, cells were pictured using an automated upright microscope system (Leica DM5500 B) coupled with Leica Cytovision software.

### 2.9. Study Population

Pathological patients in the study were women with a diagnosis of invasive ductal breast carcinoma. The subtype was assigned based on the surrogate definition of St Gallen 2013 consensus meeting guidelines [28]. All subjects were enrolled at the Ospedale Evangelico Betania (Naples, Italy). Samples processing started within 1 h of the collection, and all aliquots were stored at the SDN biobank (Naples, Italy) until use [29]. This study was approved by the Ethics Committee of IRCCS Pascale (Naples, Italy) (Protocol n. 1/16 OSS SDN). Written informed consent was obtained from all subjects. This retrospective study was conducted anonymously and conforms to the principles of the Helsinki Declaration. Both the hematoxylin and eosin and immunohistochemical (IHC) staining were made for routinely immunophenotypic parameters that were used to characterize the BC tumor subtypes utilized in this study. The clinicopathologic characteristics, tumor subtypes, and diagnosis of the patients are reported in Table 1.

### 2.10. Immunohistochemistry Assay

After surgical excision, the tissue sample was stored in 10% neutral buffered formalin for fixation and subsequent insertion in the paraffin block. Tumors that were formalin-fixed and paraffin-embedded specimens (FFPE) were sectioned and stained with the hematoxylin and eosin Thermo Scientific Gemini AS instrument to assess tissue morphology. In our study, the 17 FFPE divided into one control spleen tissue, four normal breast tissue, n°10 Luminal A BC, n°10 Luminal B BC, and n°10 HER2+ BC, were collected at the Ospedale Evangelico Betania, (Naples, Italy) and preserved at SDN Biobank (Naples, Italy) [29]. For all specimens, we selected a tumor tissue block and used it to obtain 3 µm thick sections mounted on Matsunami TOMO^®^ hydrophilic adhesion slides for our immunohistochemistry assay. Anti KCTD15 staining and the assay were performed with a Ventana BenchMark ULTRA immunostainer (Roche Diagnostics, Basel, Switzerland) using diaminobenzidine as chromogen and with reagents registered according to the manufacturer’s protocol (Ultra View Universal DAB detection kit; Ventana, Tucson, AZ, USA). The manual incubation procedure was performed using a Rabbit Anti KCTD15 antibody (Abcam Cat# ab106373), with dilution 1:400. Spleen human tissue was used as control. For the evaluation of immunohistochemical positivity, the pathologist carried out a quantitative and qualitative assessment of the intensity, extent, and intracellular distribution of the anti KCTD15 in breast cancer. The IHC intensity score of cell staining, determined as follows: 0 (low/absent staining), 1 (low/moderate staining), 2 (moderate/strong staining), 3 (strong staining), was considered as a qualitative criterion, while the percentage of positive tumor cells was considered for the quantitative criteria (cut off >10% tumor cell). In scoring KCTD15 protein expression, both the immunopositivity in the nucleus and in the cytoplasm were considered. Antigen expression was evaluated by an expert pathologist using a direct-light microscope in a bright field at 20× and 40× magnification.

## 3. Results

### 3.1. KCTD15 Is Over-Expressed in Breast Cancer HER2+

**Figure 1:**
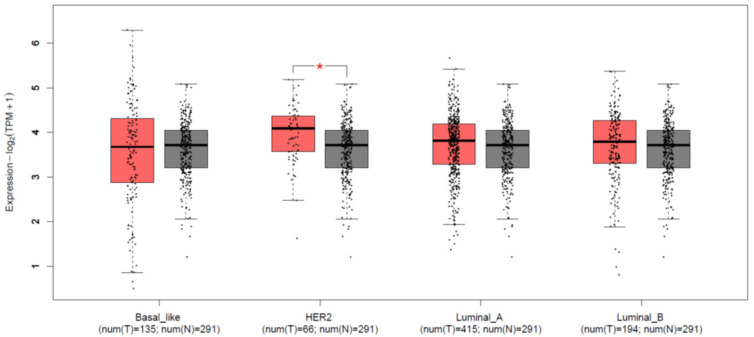
KCTD15 mRNA expression in breast cancer different subtypes and normal tissues (GEPIA). Box plots show median mRNA expression levels of KCTD15 in breast cancer tumors (red plots) and the corresponding normal tissues (gray plot). Axis units are Log2 (TPM + 1). * red = *p*-value < 0.01. T = tumor tissues. N = normal tissues.

The role and the expression levels of KCTD15 in breast cancer were preliminarily evaluated by interrogating the Cancer Genome Atlas (TCGA) and normal Genotype-Tissue Expression (GTEx) databases using the GEPIA portal to possibly highlight differences in the mRNA expression level of KCTD15 in different breast cancer subtypes. In particular, considering the heterogeneity of this cancer, we selectively focused our attention on four BC subtypes (HER2+, Triple Negative, Luminal A, and Luminal B). As shown in Figure 1, a comparative analysis of these databases indicated that the expression level of KCTD15 mRNA is significantly higher in HER2+ compared to normal tissues HER2+ (*p*-value < 0.01). Notably, we also observed that KCTD15 is the only protein of the KCTD family that is specifically over-expressed in a single BC subtype, as all other KCTD proteins have no specific BC subtype expressions. Indeed, KCTD1/KCTD5 are generally over-expressed whereas KCTD12/KCTD14 are down-regulated in all BC subtypes (Supplementary Figure S1).

**Figure 2:**
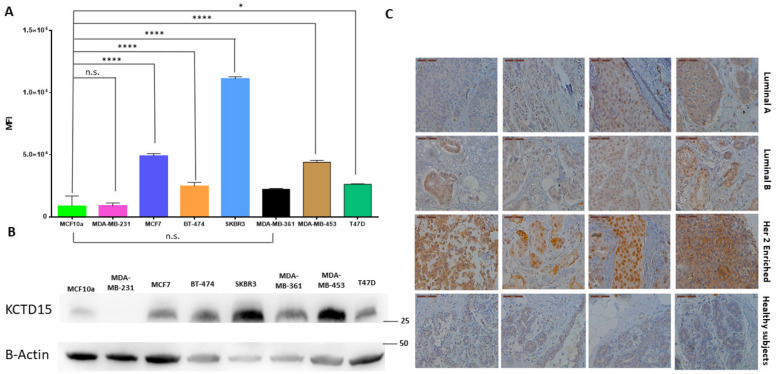
KCTD15 expression in breast cancer tissues and cell lines. (**A**) Bar-plots display fluorescence intensity (in terms of median of fluorescence and SEM) of KCTD15 in MCF10 (light green), MDA-MB-231 (pink), MCF7 (blue), BT-474 (orange), SKBR3 (light blue), MDA-MB-361 (black), MDA-MB-453 (light brown) and T47D (green). The error bar represents SEM. **** = *p* < 0.0001, * = *p* < 0.05. Mann Whitney T-test. Panel (**B**) shows KCTD15 expression using WB. Numbers represent protein marker expressed in kDa. Western blot is representative of three independent experiments with similar results. Panel (**C**) displayed KCTD15 expression level in breast tissues by IHC analysis: 4 Luminal A (score 2), 4 Luminal B (score 2), 4 HER2 enriched (score 3), and 4 healthy subtypes (score 0). Magnification 40×. Scale bars 50 µm.

**Figure 3:**
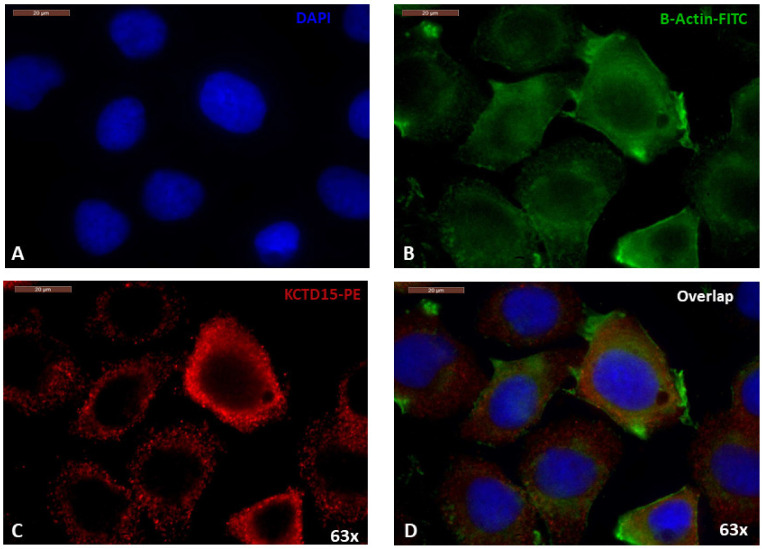
Immunofluorescence assay on SKBR3 cells. (**A**) Nuclei staining with DAPI (blue) (**B**) β-actin staining with FITC-conjugated secondary antibody. (**C**) Endogenous KCTD15 labeled with PE-conjugated secondary antibody. (**D**) Overlapping of FITC, PE, and DAPI channels. Magnification 63×. Scale bars 20 µm.

These observations, though preliminary, prompted us to evaluate the KCTD15 expression levels of the protein in model cell lines related to these BC subtypes. In particular, we monitored the protein expression levels in the cell lines that represent models for HER2+ (SKBR3 and MDA-MB-453), Triple Negative Breast Cancer (TNBC) (MDA-MB-231 and MDA-MB-361), Luminal A (MCF-7 and T47D), and Luminal B (BT474) breast cancer subtypes [30,31]. We included the MDA-MB-453 cell line among the HER2+ breast cancer models due to its peculiarity of possessing an elevated HER2+ mRNA expression combined with limited HER2 protein levels, especially when compared with those exhibited by SKBR3 [32]. The non-tumorigenic epithelial MCF10a cell line was used as a control. As shown in Figure 2A, a cytofluorimetric analysis of KCTD15 expression clearly indicates that breast cancer cell lines present significantly higher KCTD15 expression compared to MCF10A. The gating strategy for cytofluorimetric analyses was reported in Supplementary Figure S2. Within this general trend, SKBR3 presents remarkably higher values compared to the other BC-tested cell lines. This overall picture is corroborated via an analysis of the corresponding Western blot and by band quantifications (Figure 2B and Supplementary Figure S3). Based on these observations, we evaluated KCTD15 expression in the distinct BC tissue subtypes by immunohistochemistry (IHC) analyses (Figure 2C). In particular, KCTD15 expression was evaluated in Luminal A, Luminal B, and HER2+ tumor tissues extracted from 10 patients and compared with tissues obtained from healthy subjects. The protein positivity by IHC reaction was assessed by looking at the entire section of the slide. The score (absent, low, medium, or strong) was assessed by comparing the positive internal intrinsic elements of control (hyperplastic area) which show very mild positivity in the normal ductal-lining epithelium. In breast tissues derived from healthy subjects, KCTD15 is either absent or poorly expressed. Indeed, in these tissues, the observed KCTD15 levels are comparable with those observed in the basal epithelium, in adipose tissue, and in stromal cells (score 0) (Figure 2C). The expression levels of KCTD15 in four representative patients for each BC subtype are also reported in Figure 2C. Neoplastic, invasive ductal-carcinoma cells show different levels of protein expression in relation to tumor histotype. A low/moderated signal (score 1) is associated with Luminal A BC (Figure 2C, upper lane) whereas a moderate/intense staining (score 2) was detected for Luminal B BC (Figure 2C, second lane). On the other hand, a strong intensity of the signal (score 3) is associated with HER2+ breast cancer (Figure 3C, third lane) and with the positive control, i.e., IHC of anti KCTD15 antibody (Spleen tissue, Supplementary Figure S4). These findings were confirmed by the analyses of the other six patients of the cohort (Supplementary Figure S5). It is important to note that low values of KCTD15 expression, comparable to those observed in tissues isolated from healthy subjects, were observed in the portions of normal breast tissue of these BC patients (Supplementary Figure S6). Collectively, these results highlight a correlation between KCTD15 overexpression and specific BC subtypes at both at cell lines and patient-tissue levels.

### 3.2. Cellular Localization of KCTD15 in SKBR3 Cell Line

To obtain further information on the role played by KCTD15 in this context, we evaluated its localization using the immunofluorescence assay in the SKBR3 cell line that presented the highest protein-expression levels. As shown in Figure 3, the KCTD15 phycoerythrin signal overlaps with the β-actin-FITC fluorescence rather than with the nuclear signal of the DAPI. This finding is confirmed by the cytoplasmic localization of specific anti-KCTD15 antibody, highlighted by Phycoerythrin secondary antibody (Figure 3C and Supplementary Figure S7). In line with the findings reported above, no KCTD15 signal was detected in MDA-MB-231 cells that represent the negative control of immunofluorescence experiments (Supplementary Figure S8).

### 3.3. Silencing of KCTD15 Inhibits SKBR3 Proliferation and Migration, Determining a Cell Cycle Arrest

**Figure 4:**
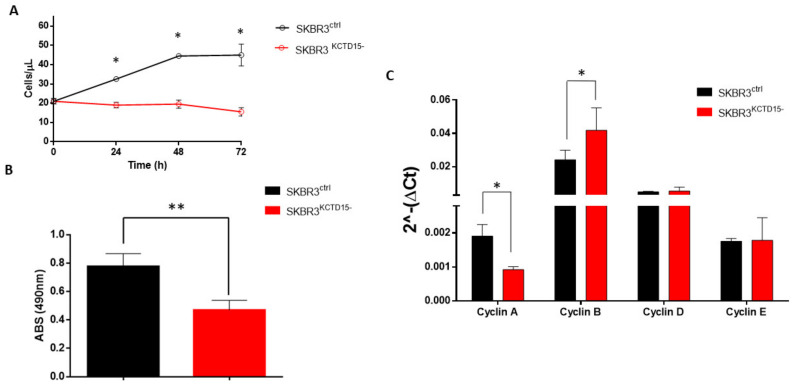
CRISPR/CAS9 KCTD15 silencing reduces SKBR3 growth. (**A**) Cell proliferation at 24, 48 and 72 h of SKBR3 control (black line) and SKBR3^KCTD15−^ (red line). Cell growth was expressed as number of cells/µL. Error bars represent the SD of three independent experiments. * = *p*-value < 0.05 (**B**) MTT assay of SKBR3^ctrl^ (Black bar) and SKBR3^KCTD15−^ (red bar) after 72 h of growth. * = *p*-value < 0.05, ** = *p*-value < 0.01, Mann–Whitney *t*-test. Error bars represent the SD of six independent experiments. (**C**) Cyclins mRNA expression levels in SKBR3^ctrl^ (black bar) and SKBR3^KCTD15−^ (red bar). The relative expression was determined using the 2−ΔCt method. Cyclins relative expression is shown as mean +/− SD of two technical independent experiments. * = *p*-value < 0.05, Mann–Whitney *t*-test.

**Figure 5:**
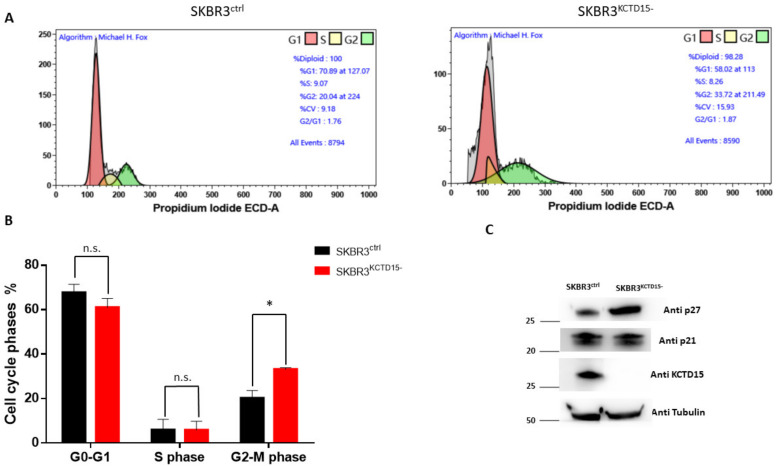
KCTD15 silencing causes the G2 m alteration of the SKBR3 cell cycle. (**A**) Flow cytometry analysis of the cell cycle distribution in SKBR3^ctrl^ (**left**) and SKBR3^KCTD15−^ (**right**) cells after 72 h of active growth. (**B**) Histogram representation of cell cycle phase percentage, derived from three independent experiments. * = *p* < 0.05 Mann–Whitney *t*-test. Error bars indicate standard deviation. (**C**) Western blot of p21, p27, KCTD15 and Tubulin in SKBR3^ctrl^ (**left**) and SKBR3^KCTD15−^ (**right**) cell extracts after 72 h of active growth. Numbers represent protein marker expressed in kDa. Western blot was repeated twice with similar results.

**Figure 6:**
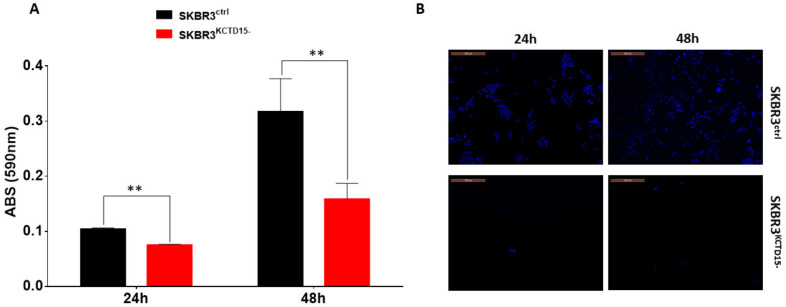
KCTD15 silencing influences the SKBR3 migration. SKBR3ctrl and SKBR3^KCTD15−^ migration was determined using trans-well chamber by Crystal violet (**A**) and DAPI (**B**) staining of migrated cells. ** = *p* < 0.01 Mann–Whitney T-test. Crystal violet signal (590 nm) is reported as mean +/− SD of three technical independent experiments. For DAPI staining, the magnification reported is 20×. Scale bars 200 µm.

Further insights into the role played by KCTD15 in BC were found by silencing the gene. The selection of clones was monitored by PCR and Western blot analysis. Clone 1 (named SKBR3^KCTD15−^) was selected because it expresses undetectable levels of KCT15 at both the gene and protein levels (Supplementary Figure S9). Moreover, no morphological significant differences were observed when comparing SKBR3^ctrl^ and SKBR3^KCTD15−^ by brightfield microscopy (Supplementary Figure S10). As shown in Figure 4A, SKBR3^KCTD15−^ cells present significantly slower growth compared to SKBR3 wild type (SKBR3^ctrl^) at 24, 48 and 72 h when a significant reduction of KCTD15 protein was detected (Supplementary Figure S11). These data were confirmed by the MTT assay conducted on SKBR3^ctrl^ and SKBR3^KCTD15−^ after 72 h of active growth (Figure 4B). The absorbance of the MTT signal decreased to approximately 50% in SKBR3^KCTD15−^, confirming that KCTD15 silencing determines a marked reduction in cell proliferation. Subsequently, since the cell growth block was not associated with evident mortality of the SKBR3^KCTD15−^ cells after Propidium Iodide staining (Supplementary Figure S12), we characterized the effect of KCTD15 silencing on the cell cycle. We evaluated cyclin A, cyclin B, cyclin D, and cyclin E mRNA expression in SKBR3^ctrl^ and SKBR3^KCTD15−^ cells. As shown in Figure 4C, we did not find significant differences for cyclin D and cyclin E. On the contrary, we observed statistically significant differences for cyclin A and cyclin B. Therefore, in SKBR3^KCTD15−^, an increase in the mRNA expression of cyclin B, associated with a reduction of cyclin A expression, determines a possible block in the G2/M phase of the cell cycle, thereby explaining the proliferative inhibition. To further confirm this hypothesis, we analyzed the cell cycle by FCM during a 72 h period of active growth (Figure 5A,B and Supplementary Figure S1). Although no significant difference was detected in the initial phases of the active growth (T0–48 h, Supplementary Figure S13 panel A–C), a significant rise in the G2-M phase occurs in SKBR3^KCTD15−^ cells when compared to SKBR3^ctrl^ cells after 72 h of active growth (Figure 5C). Furthermore, the block of the cell cycle at the G2-M phase was confirmed by WB analysis of p27 protein, an inhibitor of cyclin A [33]. As illustrated in Figure 5C, a significant increase in p27 expression occurs in SKBR3^KCTD15−^ cells that likely determines the reduction of cyclin A expression. At the same time, in SKBR3^KCTD15−^ cells, no variation compared to the control cell line was detected for p21 expression, which is an inhibitor of cyclin D and E [34]. In addition, we evaluated the migration capability of SKBR3^KCTD15−^ cells in comparison with the control. As observed in Figure 6, a significant reduction of SKBR3 cell migration occurs when KCTD15 is silenced. Indeed, we observed a reduction of about 20% in terms of cell migration after just 24 h of incubation and of more than 50% after 48 h of incubation (Figure 6A). This quantitative observation was confirmed by the DAPI staining of migrated cells, which is suggestive of a strong reduction in the migration capacity of cells deprived of the KCTD15 gene (Figure 6B).

### 3.4. Silencing of KCTD15 Sensitizes SKBR3 Cells to Doxorubicin and Trastuzumab Treatments

**Figure 7:**
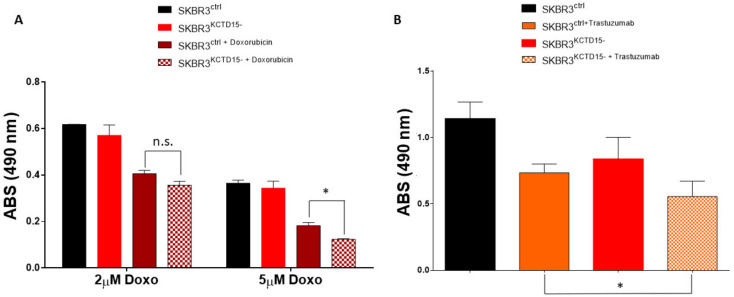
KCTD15 silencing increase the Doxorubicin and Trastuzumab sensitivity of SKBR3 cells. (**A**) MTT assay of untreated SKBR3ctrl and SKBR3^KCTD15−^ (filled bars) and doxorubicin treated SKBR3^ctrl^ and SKBR3^KCTD15−^ (squared bars) at 2 and 5 μM for 16h. * = *p*-value < 0.05. (**B**) MTT assay of Trastuzumab treated SKBR3^ctrl^ (orange bar) and SKBR3^KCTD15−^ (orange square bar) in comparison with the untreated control cells (SKBR3^ctrl^, Black bar and SKBR3^KCTD15−^, Red bar). * = *p*-value < 0.05, Mann–Whitney *t*-test. n.s. = not significative. Error bars represent SD of three independent experiments.

**Figure 8:**
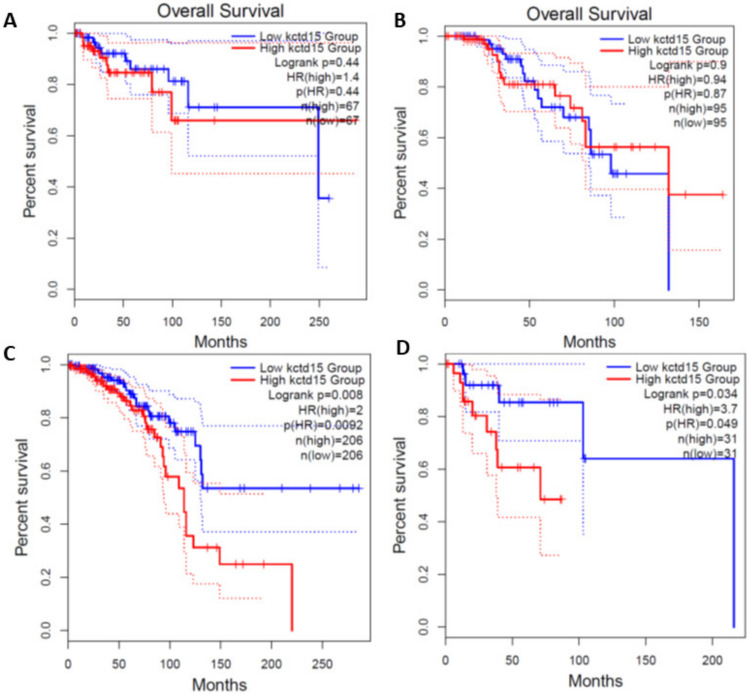
KCTD15 mRNA predicts worst survival in breast cancer subtypes. Kaplan–Meier plots generated from GEPIA show Overall Survival in breast cancer subtypes with high and low levels of KCTD15 mRNA. (**A**) Triple Negative patients. (**B**) Luminal B patients. (**C**) Luminal A patients. (**D**) HER2+ patients. Log-rank, Chi-squared test for statistical analysis.

To evaluate whether KCTD15 silencing could improve the action of drugs commonly used in the treatment of breast cancer, we decided to estimate the in vitro effect of Doxorubicin, a generic chemotherapeutic agent [31], and the Trastuzumab, a drug specifically used in the treatment of HER2+ breast cancer patients [35]. We treated SKBR3^KCTD15−^ and the SKBR3^ctrl^ cells with 2 and 5 µM of doxorubicin for 16 h. As shown in Figure 7A, 2 µM doxorubicin is more effective on SKBR3^KCTD15−^ cells than SKBR3^ctrl^ cells. Furthermore, by increasing the concentration of doxorubicin to 5 µM, the effects on cell metabolism become significantly greater than the control cells. Furthermore, as shown in Figure 7B, the silencing of KCTD15 sensitized the SKBR3 cells to Trastuzumab, showing a significantly greater effect for the HER2+ cell line compared to SKBR3 cells treated with Trastuzumab alone. Moreover, we also examined overall survival (OS) in breast cancer patients who received treatment, either chemical or hormonal, using the Kaplan–Meier plot as a function of KCTD15 expression levels. We analyzed breast cancer patient data from the TCGA database using the GEPIA portal. As shown in Figure 8, there were no significant differences between Triple Negative and Luminal B breast cancer patients in terms of OS, which was determined by dividing the patients into the two subgroups (low and high KCTD15 expression, Figure 8A,B). On the other hand, significant differences are reported for Luminal A and HER2+ breast cancer patients (Figure 8C,D). These results suggest that high mRNA expression levels of KCTD15 are associated with a poorer survival of patients in a subtype-specific manner.

## 4. Discussion

Breast cancer classification is based on the presence of specific receptors at the cellular membrane level of BC cells; the most important receptors involved in the iper-activation of proliferation pathways are ER, PR and HER2+ [36]. Luminal A (ER+; PR+; HER2−) BC subtype progression is supported by deregulated ER activation via the binding of its ligand, estrogen, or by estrogen-independent receptor activation by Tyrosine Kinase Receptors (RTKs), including Epidermal Growth Factor (EGFR), HER2, Insulin-like Growth Factor 1 (IGF-1R), PI3K/Akt/mTOR, or MAPK1. ER activation can stimulate pathways involved in cell proliferation and block apoptosis [37,38,39]. Luminal B breast cancer (ER+; PR+; HER2+) is supported by a proliferative stimulus triggered not only by the activation of the ER pathway but also by the stimulation of the HER2-mediated signaling pathway [36]. This signaling pathway becomes prominent in the HER2+ BC subtype (ER−; PR−; HER2+) where the overexpression of the HER2 receptor at the level of the plasma membrane of the breast cancer cells is evident [40]. Triple-negative breast cancer (ER−; PR−; HER2−), whose biology is poorly understood, presents a higher pathologic grade compared to the other subtypes [41]. In the present study, we show that KCTD15 is significantly upregulated in tissues extracted from patients affected by different BC subtypes and in the related cell models. The levels of the protein are, however, differentiated in the tissues of patients affected by distinct BC subtypes. Indeed, KCTD15 appears to be highly overexpressed in HER2+ BC tissues, whereas it is only moderately detectable or even absent in the other BC subtypes investigated here (Luminal A, Luminal B, and Triple Negative). Since database analyses indicate that KCTD15 is the only member of the KCTD family to be overexpressed in a BC subtype, we evaluated the protein expression levels in BC cell models by cytofluorimetric and Western blot analyses. The overexpression of the protein was particularly evident in SKBR3 cells, a human breast cancer cell line that overexpresses the HER2 (Neu/ErbB-2) gene product, where its silencing reduced cell proliferation associated with a block of cell cycle progression in the G2/M phase due to the upregulation of p27, a cell-cycle inhibitor [33]. In HER2+ BC, the NF-κB pathway is iper-activated [42], and the present findings are in line with the recent association between KCTD15 overexpression and NF-κB activation through the upregulation of IKK-β phosphorylation [19]. Interestingly, it has been reported that the IKK-β/NF-κB mediates the degradation of p27 [43]. Collectively, the literature data and the present findings suggest that KCTD15 may favor cell cycle progression through the activation of IKK-β which, in turn, downregulates p27.

Interestingly, we found that KCTD15 silencing makes the HER2+ cell line SKBR3 more sensitive to treatment with different drugs. Upon KCTD15 silencing, an increment of the chemotherapeutic effect of doxorubicin, a generic chemotherapy drug, and of Trastuzumab, an innovative monoclonal antibody widely used in HER2+ BC, was observed. In particular, KCTD15 silencing in combination with the use of doxorubicin induces a significant decrease in SKBR3 metabolism. This finding opens pathways to the development of potential therapeutic approaches that could also be tested on trastuzumab-resistant cells [44,45]. Indeed, the use of trastuzumab or doxorubicin in combination with agents that are able to inactivate KCTD15 could increase the success rate of HER2+ treatments. In this context, it worth noting that KCTDs, and KCTD15 specifically, are typically well-folded and likely druggable proteins, and peptides targeting these proteins have already been reported in the literature [46,47,48]. Data obtained in this study converge to underline a stronger role of the KCTD15 protein in the HER2+ compared to the BC subtypes. These in vitro results are suggestive of a role of the KCTD15 protein in cell proliferation mediated by the tHER2 receptor. Moreover, the present findings suggest that the monitoring and the modulation of KCTD15 expression and activity can be effectively used for both diagnostic/prognostic and therapeutic scopes with a specific focus on HER2+ BC. Finally, considering the involvement of other KCTD proteins in BC [10,49,50,51], investigations aimed at elucidating precise molecular mechanisms involving KCTD15 are in progress. The ability of this protein to play roles in diversified contexts suggests that it likely performs a basic biochemical activity.

## Figures and Tables

**Table 1:** Clinico-pathological characteristics of the study patients.

Healthy Control Sample (*n* = 4)	
34–55	(42.5)
**Breast Cancer Sample (*n* = 30)**	
39–87	(65)
**Sex**	
Woman	34
Man	0
**Histologic Types**	
Invasive Ductal Carcinoma	30
Hyperplasia	2
Gynecomastia	2
**Subtype**	
Luminal A	10
Luminal B	10
HER2 +	10
**Ki67**	
Low (0–29%)	20
High (30–100%)	14
**Grade**	
G1	1
G2	18
G3	11
**Tumor size**	
(0.1–2 cm)	20
(2–5 cm)	10
>5cm	0
**Lymph node Status (N)**	
Involved	10
Uninvolved	20

## Data Availability

Gepia portal was used to obtain data from public database (http://gepia.cancer-pku.cn/, last viewed date: 23 November 2020).

## Supplementary Materials

The following are available online at www.mdpi.com/article/10.3390/diagnostics12030591/s1.

## Abbreviations

BC	Breast Cancer
ER	estrogen receptor
PR	progesterone receptor
GTEx	Genotype-Tissue Expression
HER2	human epidermal growth factor receptor 2
KCTD	potassium channel tetramerization domain
IHC	Immunohistochemistry
MFI	Mean Fluorescence Intensity
FCM	Flow CytoMetry
OS	Overall Survival
FFPE	Formalin-Fixed and Paraffin-Embedded
TNBC	Triple-negative breast cancer
TCGA	Cancer Genome Atlas
RTKs	Tyrosine Kinase Receptors
EGFR	Epidermal Growth Factor
IGF-1R	Insulin-like Growth Factor 1

## References

[1] Skoblov M., Marakhonov A., Marakasova E., Guskova A., Chandhoke V., Birerdinc A., Baranova A. (2013). Protein partners of KCTD proteins provide insights about their functional roles in cell differentiation and vertebrate development. BioEssays News Rev. Mol. Cell. Dev. Biol..

[2] Teng X., Aouacheria A., Lionnard L., Metz K.A., Soane L., Kamiya A., Hardwick J.M. (2019). KCTD: A new gene family involved in neurodevelopmental and neuropsychiatric disorders. CNS Neurosci. Ther..

[3] Canettieri G., Di Marcotullio L., Greco A., Coni S., Antonucci L., Infante P., Pietrosanti L., De Smaele E., Ferretti E., Miele E. (2010). Histone deacetylase and Cullin3–RENKCTD11 ubiquitin ligase interplay regulates Hedgehog signalling through Gli acetylation. Nat. Cell Biol..

[4] Zheng S., Abreu N., Levitz J., Kruse A.C. (2019). Structural basis for KCTD-mediated rapid desensitization of GABAB signalling. Nature.

[5] Zhong Y., Yang J., Xu W.W., Wang Y., Zheng C.-C., Li B., He Q.-Y. (2017). KCTD12 promotes tumorigenesis by facilitating CDC25B/CDK1/Aurora A-dependent G2/M transition. Oncogene.

[6] Zhao J., Bradfield J.P., Li M., Wang K., Zhang H., Kim C.E., Annaiah K., Glessner J., Thomas K., Garris M. (2009). The Role of Obesity-associated Loci Identified in Genome-wide Association Studies in the Determination of Pediatric BMI. Obes. Silver Spring Md..

[7] Smaldone G., Balasco N., Pirone L., Caruso D., Di Gaetano S., Pedone E.M., Vitagliano L. (2019). Molecular basis of the scalp-ear-nipple syndrome unraveled by the characterization of disease-causing KCTD1 mutants. Sci. Rep..

[8] Spiombi E., Angrisani A., Fonte S., De Feudis G., Fabretti F., Cucchi D., Izzo M., Infante P., Miele E., Po A. (2019). KCTD15 inhibits the Hedgehog pathway in Medulloblastoma cells by increasing protein levels of the oncosuppressor KCASH2. Oncogenesis.

[9] Ye R., Kuang X., Zeng H., Shao N., Lin Y., Wang S. (2020). KCTD12 promotes G1/S transition of breast cancer cell through activating the AKT/FOXO1 signaling. J. Clin. Lab. Anal..

[10] Angrisani A., Di Fiore A., De Smaele E., Moretti M. (2021). The emerging role of the KCTD proteins in cancer. Cell Commun. Signal..

[11] Li X., Chen C., Wang F., Huang W., Liang Z., Xiao Y., Wei K., Wan Z., Hu X., Xiang S. (2014). KCTD1 Suppresses Canonical Wnt Signaling Pathway by Enhancing β-catenin Degradation. PLoS ONE.

[12] Dutta S., Dawid I.B. (2010). Kctd15 inhibits neural crest formation by attenuating Wnt/β-catenin signaling output. Dev. Camb. Engl..

[13] Gamero-Villarroel C., González L.M., Rodríguez-López R., Albuquerque D., Carrillo J.A., García-Herráiz A., Flores I., Gervasini G. (2017). Influence ofTFAP2BandKCTD15genetic variability on personality dimensions in anorexia and bulimia nervosa. Brain Behav..

[14] Pirone L., Smaldone G., Spinelli R., Barberisi M., Beguinot F., Vitagliano L., Miele C., Di Gaetano S., Raciti G.A., Pedone E. (2019). KCTD1: A novel modulator of adipogenesis through the interaction with the transcription factor AP2α. Biochim. Biophys. Acta (BBA) Mol. Cell Biol. Lipids.

[15] Smaldone G., Pirone L., Capolupo A., Vitagliano L., Monti M.C., Di Gaetano S., Pedone E. (2018). The essential player in adipogenesis GRP78 is a novel KCTD15 interactor. Int. J. Biol. Macromol..

[16] Marneros A.G., Beck A.E., Turner E.H., McMillin M.J., Edwards M.J., Field M., Sobreira N.L.D.M., Perez A.B.A., Fortes J.A., Lampe A.K. (2013). Mutations in KCTD1 Cause Scalp-Ear-Nipple Syndrome. Am. J. Hum. Genet..

[17] Smaldone G., Coppola L., Incoronato M., Parasole R., Ripaldi M., Vitagliano L., Mirabelli P., Salvatore M. (2020). KCTD15 Protein Expression in Peripheral Blood and Acute Myeloid Leukemia. Diagnostics.

[18] Smaldone G., Beneduce G., Incoronato M., Pane K., Franzese M., Coppola L., Cordella A., Parasole R., Ripaldi M., Nassa G. (2019). KCTD15 is overexpressed in human childhood B-cell acute lymphoid leukemia. Sci. Rep..

[19] Smaldone G., Coppola L., Pane K., Franzese M., Beneduce G., Parasole R., Menna G., Vitagliano L., Salvatore M., Mirabelli P. (2021). KCTD15 deregulation is associated with alterations of the NF-κB signaling in both pathological and physiological model systems. Sci. Rep..

[20] Carey L.A., Perou C.M., Livasy C.A., Dressler L.G., Cowan D., Conway K., Karaca G., Troester M.A., Tse C.K., Edmiston S. (2006). Race, Breast Cancer Subtypes, and Survival in the Carolina Breast Cancer Study. JAMA J. Am. Med Assoc..

[21] Sorlie T., Perou C.M., Tibshirani R., Aas T., Geisler S., Johnsen H., Hastie T., Eisen M.B., van de Rijn M., Jeffrey S.S. (2001). Gene expression patterns of breast carcinomas distinguish tumor subclasses with clinical implications. Proc. Natl. Acad. Sci. USA.

[22] Greenwalt I., Zaza N., Das S., Li B.D. (2020). Precision Medicine and Targeted Therapies in Breast Cancer. Surg. Oncol. Clin. N. Am..

[23] Hsu P.D., Lander E.S., Zhang F. (2014). Development and Applications of CRISPR-Cas9 for Genome Engineering. Cell.

[24] Györffy B., Lanczky A., Eklund A.C., Denkert C., Budczies J., Li Q., Szallasi Z. (2009). An online survival analysis tool to rapidly assess the effect of 22,277 genes on breast cancer prognosis using microarray data of 1809 patients. Breast Cancer Res. Treat..

[25] Tang Z., Kang B., Li C., Chen T., Zhang Z. (2019). GEPIA2: An enhanced web server for large-scale expression profiling and interactive analysis. Nucleic Acids Res..

[26] Canonici A., Gijsen M., Mullooly M., Bennett R., Bouguern N., Pedersen K., O’Brien A.N., Roxanis I., Li J.-L., Bridge E. (2013). Neratinib overcomes trastuzumab resistance in HER2 amplified breast cancer. Oncotarget.

[27] Fox M.H. (1980). A model for the computer analysis of synchronous DNA distributions obtained by flow cytometry. Cytometry.

[28] Untch M., Gerber B., Harbeck N., Jackisch C., Marschner N., Möbus V., Von Minckwitz G., Loibl S., Beckmann M.W., Blohmer J.-U. (2013). 13th st. Gallen international breast cancer conference 2013: Primary therapy of early breast cancer evidence, controversies, consensus—Opinion of a german team of experts (zurich 2013). Breast Care Basel Switz..

[29] Mirabelli P., Incoronato M., Coppola L., Infante T., Parente C.A., Nicolai E., Soricelli A., Salvatore M. (2017). SDN Biobank: Bioresource of Human Samples Associated with Functional and/or Morphological Bioimaging Results for the Study of Oncological, Cardiological, Neurological, and Metabolic Diseases. Open J. Bioresour..

[30] Neve R.M., Chin K., Fridlyand J., Yeh J., Baehner F.L., Fevr T., Clark L., Bayani N., Coppe J.-P., Tong F. (2006). A collection of breast cancer cell lines for the study of functionally distinct cancer subtypes. Cancer Cell.

[31] Shi Y., Yu Y., Wang Z., Wang H., Bieerkehazhi S., Zhao Y., Suzuk L., Zhang H. (2016). Second-generation proteasome inhibitor carfilzomib enhances doxorubicin-induced cytotoxicity and apoptosis in breast cancer cells. Oncotarget.

[32] Vranic S., Gatalica Z., Wang Z.-Y. (2011). Update on the molecular profile of the MDA-MB-453 cell line as a model for apocrine breast carcinoma studies. Oncol. Lett..

[33] Abbastabar M., Kheyrollah M., Azizian K., Bagherlou N., Tehrani S.S., Maniati M., Karimian A. (2018). Multiple functions of p27 in cell cycle, apoptosis, epigenetic modification and transcriptional regulation for the control of cell growth: A double-edged sword protein. DNA Repair.

[34] Karimian A., Ahmadi Y., Yousefi B. (2016). Multiple functions of p21 in cell cycle, apoptosis and transcriptional regulation after DNA damage. DNA Repair.

[35] Bradley R., Braybrooke J., Gray R., Hills R., Liu Z., Peto R., Davies L., Dodwell D., McGale P., Pan H. (2021). Trastuzumab for early-stage, HER2-positive breast cancer: A meta-analysis of 13 864 women in seven randomised trials. Lancet Oncol..

[36] Eroles P., Bosch A., Pérez-Fidalgo J.A., Lluch A. (2012). Molecular biology in breast cancer: Intrinsic subtypes and signaling pathways. Cancer Treat. Rev..

[37] Osborne C.K., Schiff R. (2011). Mechanisms of Endocrine Resistance in Breast Cancer. Annu. Rev. Med..

[38] Baselga J. (2011). Targeting the Phosphoinositide-3 (PI3) Kinase Pathway in Breast Cancer. Oncologist.

[39] Tokunaga E., Hisamatsu Y., Tanaka K., Yamashita N., Saeki H., Oki E., Kitao H., Maehara Y. (2014). Molecular mechanisms regulating the hormone sensitivity of breast cancer. Cancer Sci..

[40] Mitri Z., Constantine T., O’Regan R. (2012). The HER2 Receptor in Breast Cancer: Pathophysiology, Clinical Use, and New Advances in Therapy. Chemother. Res. Pract..

[41] Sharma P. (2016). Biology and Management of Patients with Triple-Negative Breast Cancer. Oncologist.

[42] Merkhofer E.C., Cogswell P., Baldwin A.S. (2009). Her2 activates NF-κB and induces invasion through the canonical pathway involving IKKα. Oncogene.

[43] Guo W., Liu J., Jian J., Li J., Wan Y., Huang C. (2014). IKK-β/NF-κB p65 mediates p27Kip1 protein degradation in arsenite response. Biochem. Biophys. Res. Commun..

[44] Li X., Xu J., Tang X., Liu Y., Yu X., Wang Z., Liu W. (2016). Anthocyanins inhibit trastuzumab-resistant breast cancer in vitro and in vivo. Mol. Med. Rep..

[45] Sajadimajd S., Yazdanparast R. (2015). Differential behaviors of trastuzumab-sensitive and -resistant SKBR3 cells treated with menadione reveal the involvement of Notch1/Akt/FOXO1 signaling elements. Mol. Cell. Biochem..

[46] de Paola I., Pirone L., Palmieri M., Balasco N., Esposito L., Russo L., Mazzà D., Di Marcotullio L., Di Gaetano S., Malgieri G. (2015). Cullin3—BTB Interface: A Novel Target for Stapled Peptides. PLoS ONE.

[47] Liu Z., Xiang Y., Sun G. (2013). The KCTD family of proteins: Structure, function, disease relevance. Cell Biosci..

[48] Balasco N., Pirone L., Smaldone G., Di Gaetano S., Esposito L., Pedone E.M., Vitagliano L. (2014). Molecular recognition of Cullin3 by KCTDs: Insights from experimental and computational investigations. Biochim. Biophys. Acta (BBA) Proteins Proteom..

[49] Murakami A., Maekawa M., Kawai K., Nakayama J., Araki N., Semba K., Taguchi T., Kamei Y., Takada Y., Higashiyama S. (2018). Cullin-3/KCTD10 E3 complex is essential for Rac1 activation through RhoB degradation in human epidermal growth factor receptor 2-positive breast cancer cells. Cancer Sci..

[50] Grinchuk O.V., Motakis E., Kuznetsov V.A. (2010). Complex sense-antisense architecture of TNFAIP1/POLDIP2 on 17q11.2 represents a novel transcriptional structural-functional gene module involved in breast cancer progression. BMC Genom..

[51] Canales J., Cruz P., Díaz N., Riquelme D., Leiva-Salcedo E., Cerda O. (2020). K^+^ Channel Tetramerization Domain 5 (KCTD5) Protein Regulates Cell Migration, Focal Adhesion Dynamics and Spreading through Modulation of Ca^2+^ Signaling and Rac1 Activity. Cells.

