# Supplementary material for: KCTD15 Is Overexpressed in her2+ Positive Breast Cancer Patients and Its Silencing Attenuates Proliferation in SKBR3 CELL LINE
Source: Diagnostics (Basel). 2022 Feb 25;12(3):591. doi: 10.3390/diagnostics12030591 (PMC8947324; doi:10.3390/diagnostics12030591)
Supplement: Supplementary file 1 [file diagnostics-12-00591-s001.zip › diagnostics-1577425-supplementary.pdf]

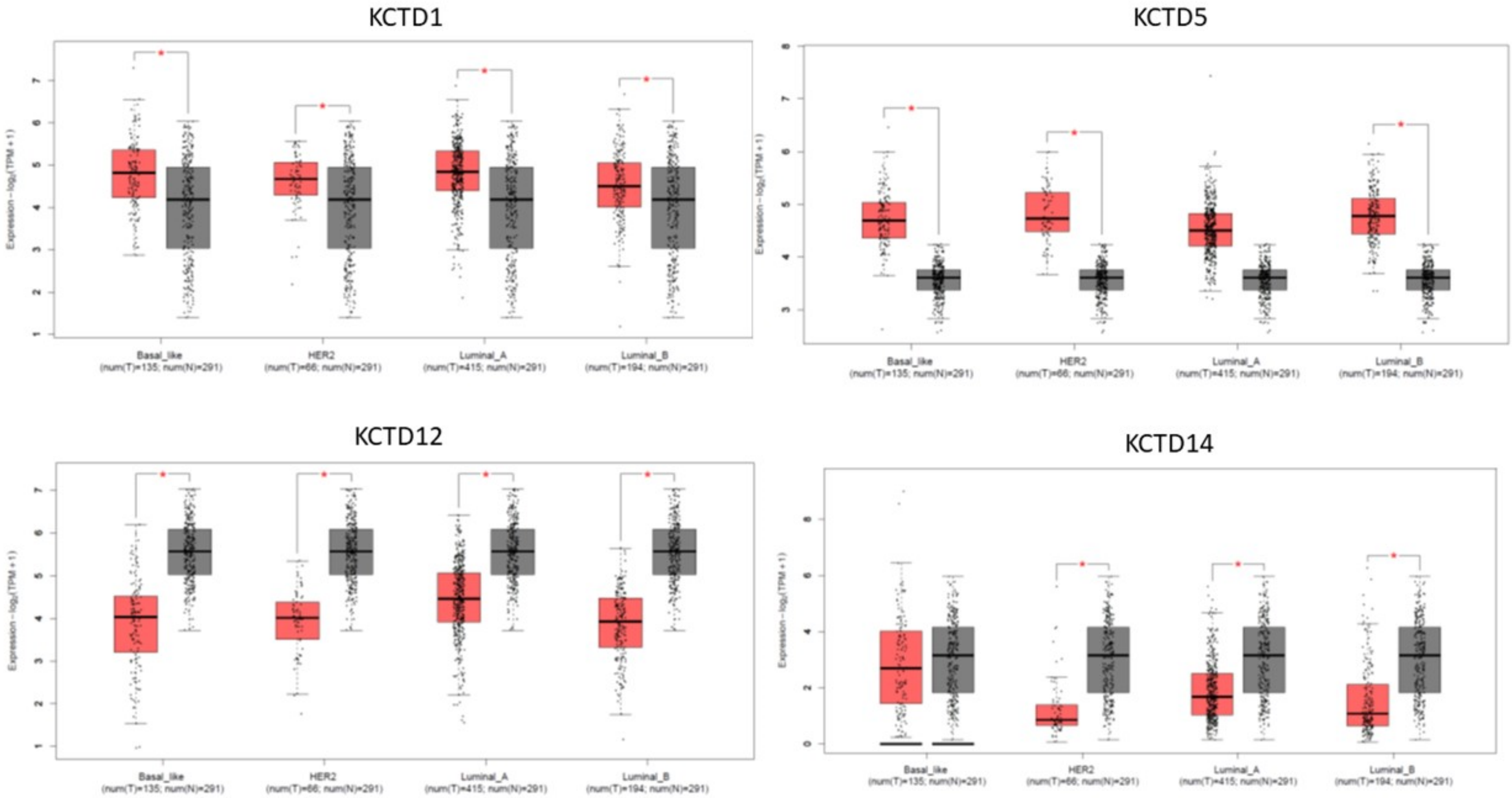

Supplementary Figure S1. KCTDs mRNA expression in breast cancer different subtypes and normal tissues. (GEPIA). Box plots show median mRNA expression levels of KCTD1, KCTD5 (upper panels), KCTD12, and KCTD14 (lower panels) in breast cancer tumors (red plots) and the corresponding normal tissues (gray plot).

Axis units are Log2 (TPM + 1). *=p-value < 0.01. T= tumor tissues. N= normal tissues

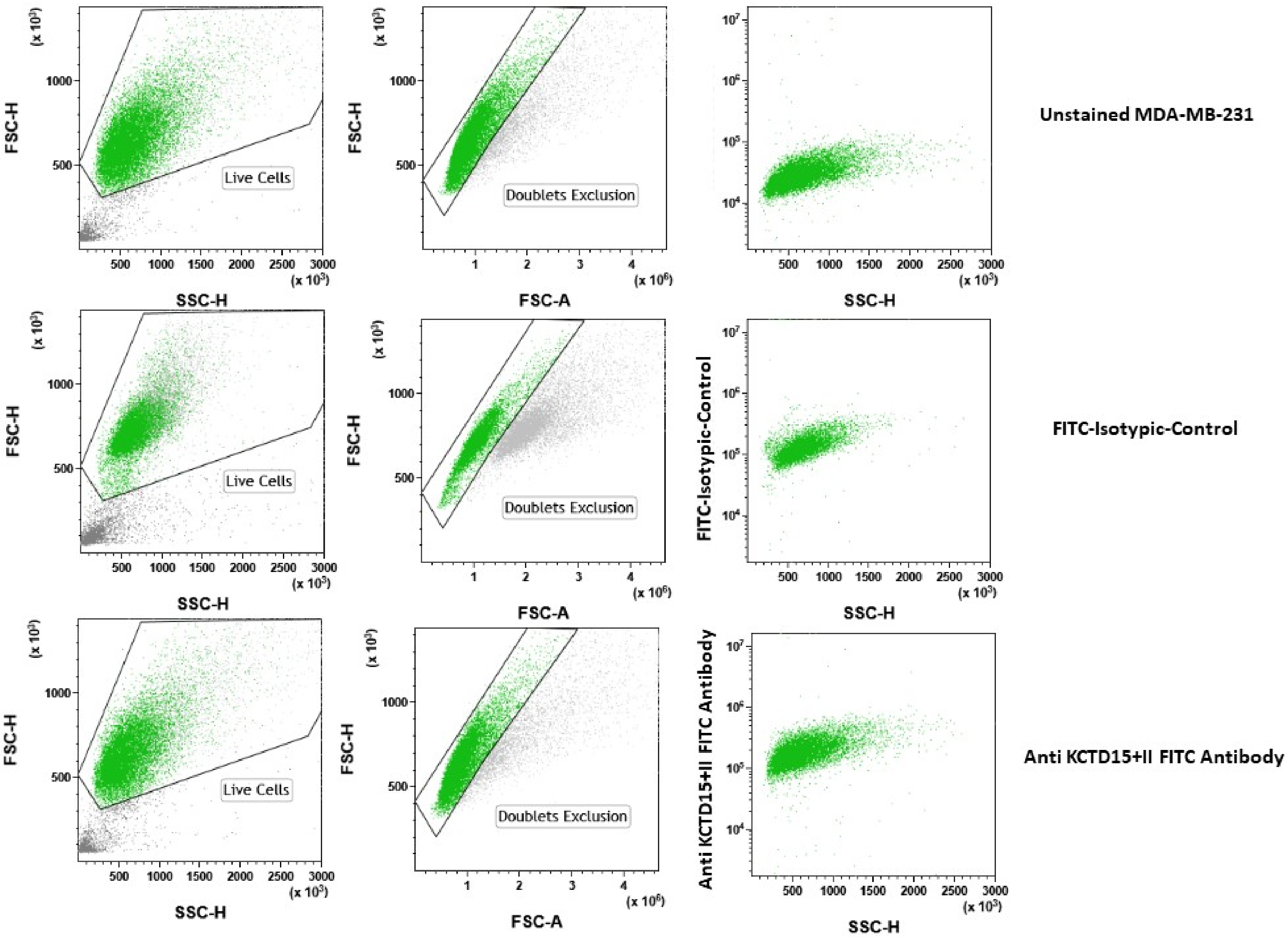

Supplementary Figure S2. Gating strategy. Representative used gating strategy for MDA-MB-231 for cytofluorimetric KCTD15 detection. The left and middle panels show the selection of live cells and the doublets exclusion, respectively. The right panels reports the fluorescence signal of unstained cells (upper), FITC-Isotypic antibody control (middle) and the anti KCTD15 mouse monoclonal antibody and secondary antimouse FITC conjugated antibody (lower panel).

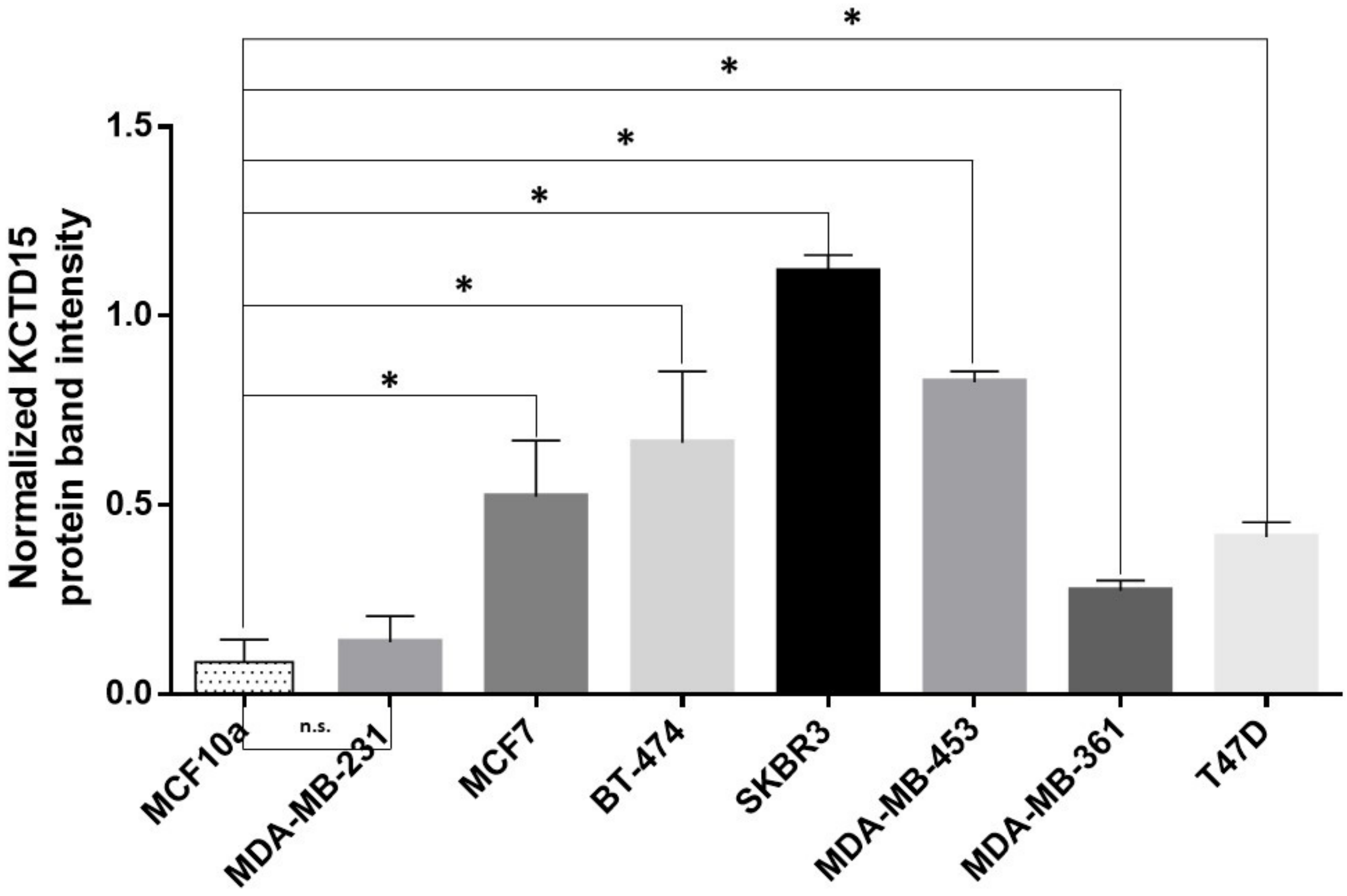

Supplementary Figure S3. KCTD15 protein band quantification. WB protein band quantification of KCTD15 in different breast cancer cell lines. *= Mann-Whithey unpaired T-Test. SD represents protein band quantification derived from three independent experiments. n.s.=not significative.

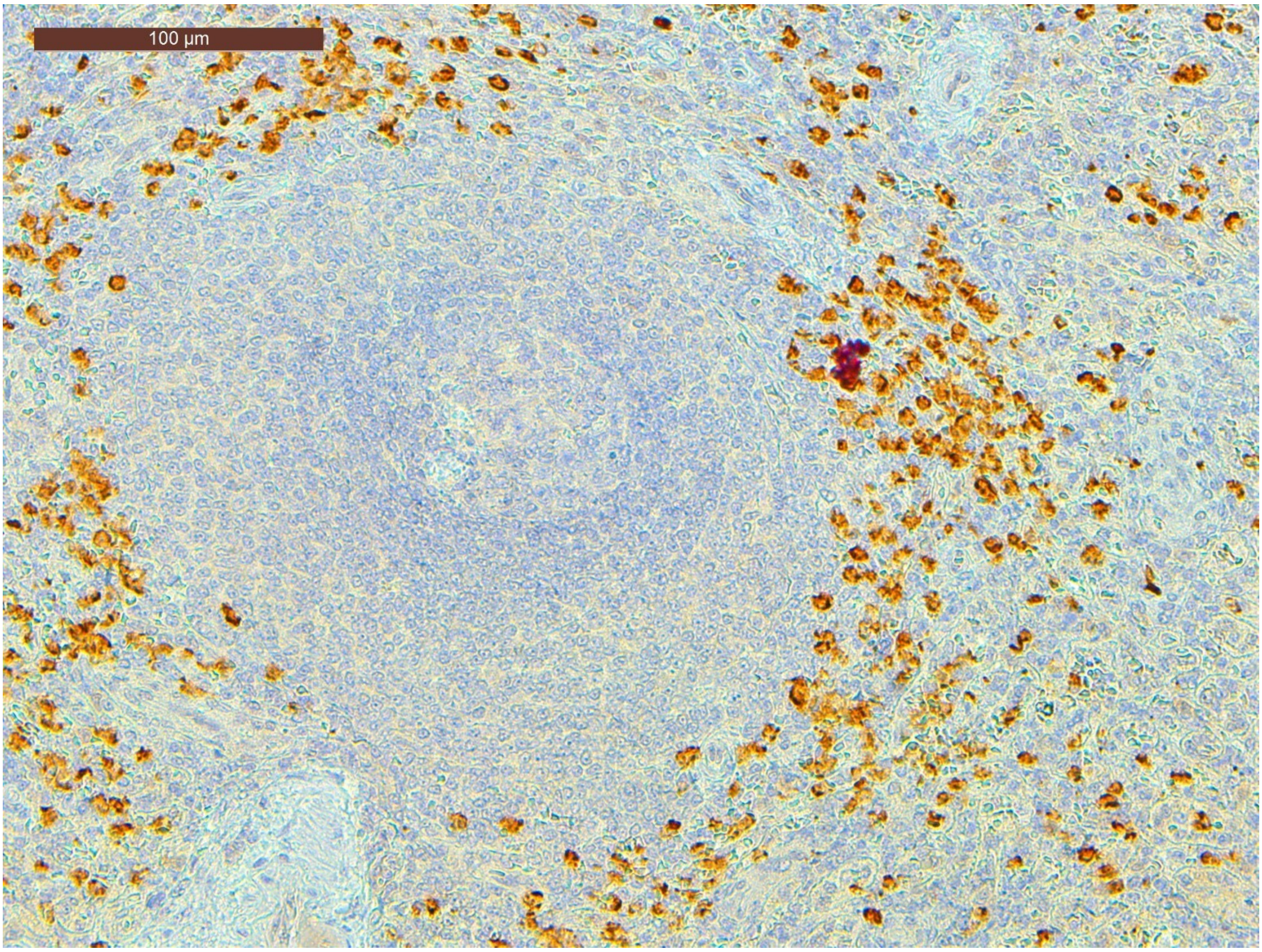

Supplementary Figure S4. KCTD15 IHC analysis of Spleen Tissue. KCTD15 expression level in Spleen tissues used as IHC positive control as reported in antibody datasheet. Magnification 40x. Scale Bar 100µm.

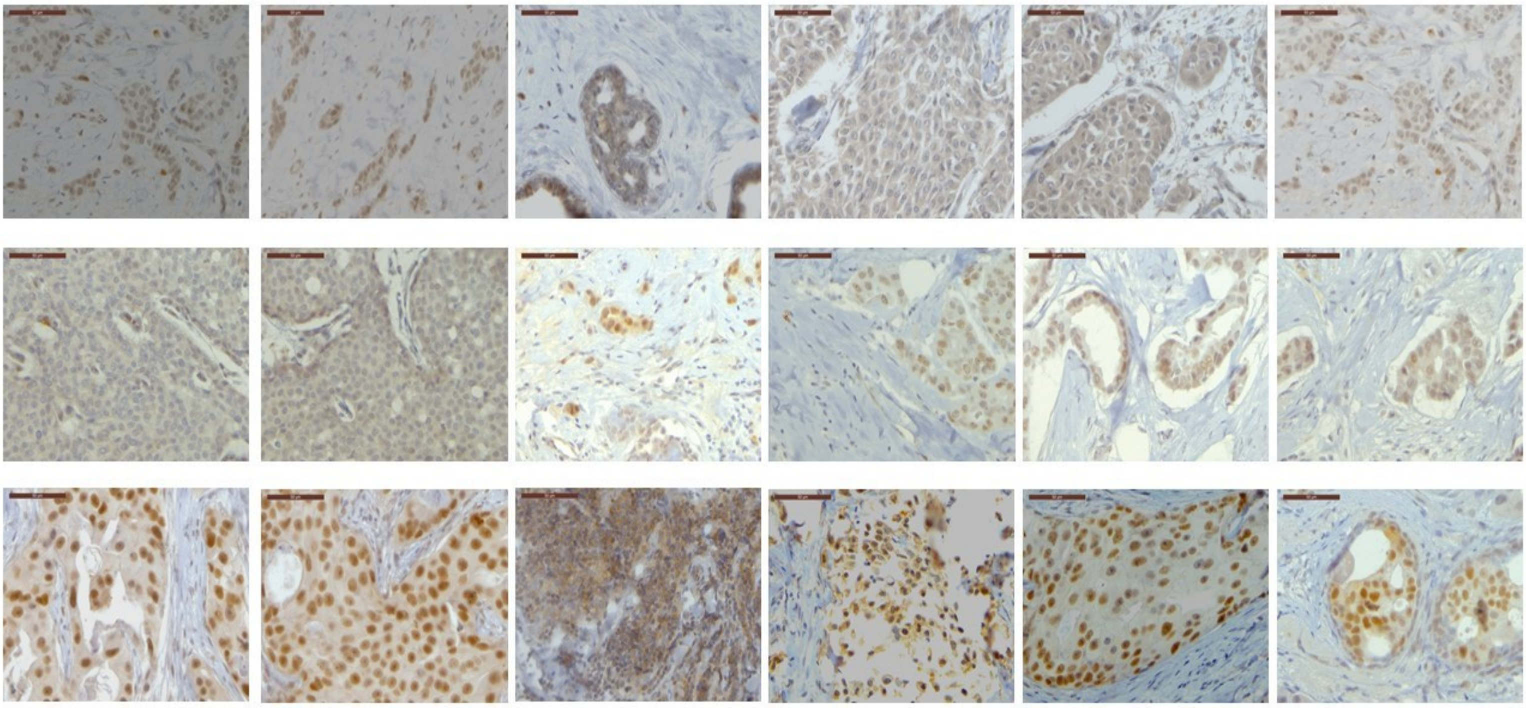

Supplementary Figure S5. KCTD15 IHC analyses . KCTD15 expression level in breast tissues by IHC analysis: 6 Luminal A (score 2, upper lane), 6 Luminal B (score 2, middle lane), 6 HER2 enriched (score 3, lower lane).

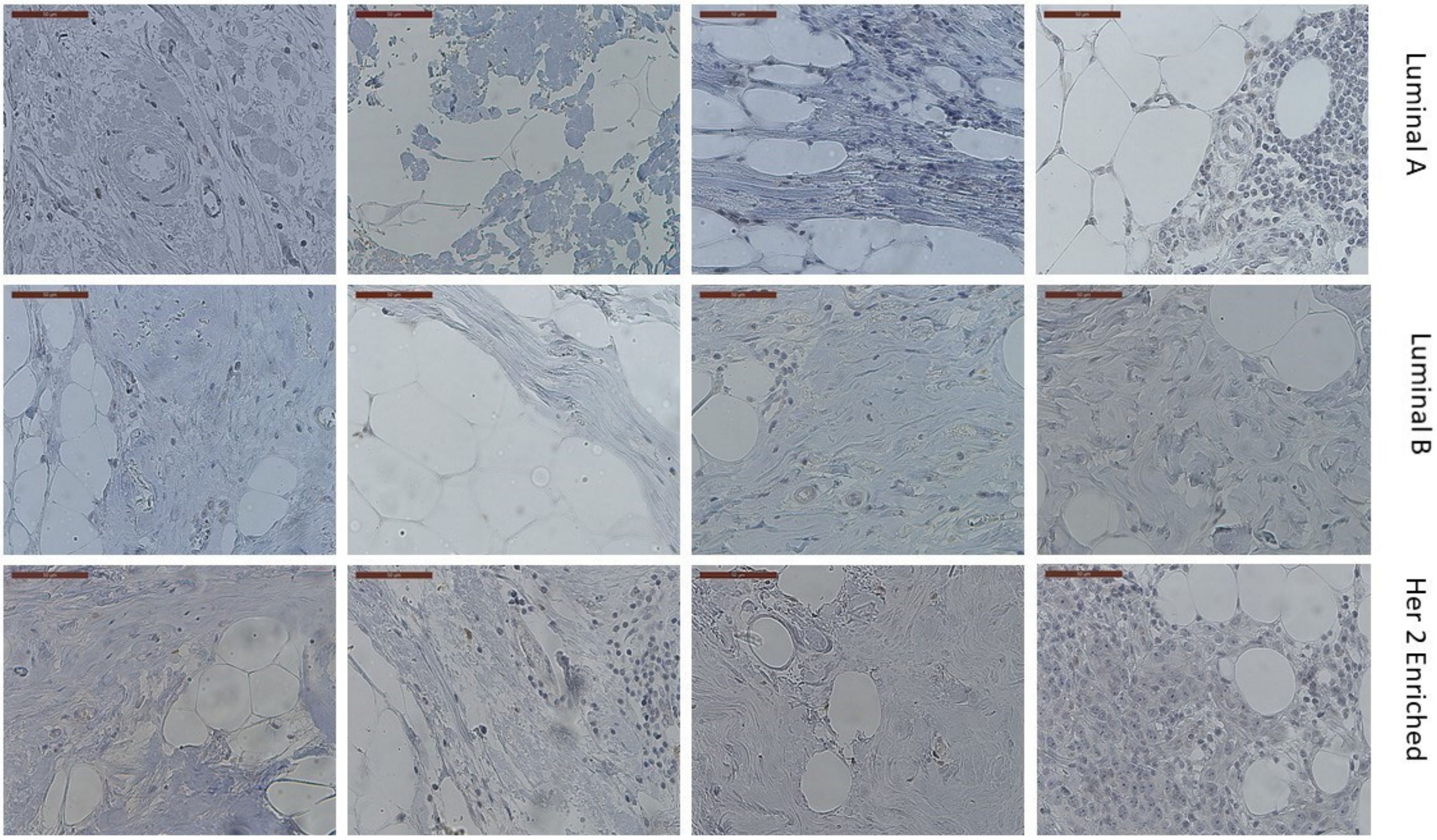

Supplementary Figure S6. KCTD15 IHC analysis of region not invaded by neoplasm. KCTD15 expression level in the region not invaded by neoplasm derived from the same patients reported in Figure 2-C. Magnification 40 × . Scale bars 50 µm.

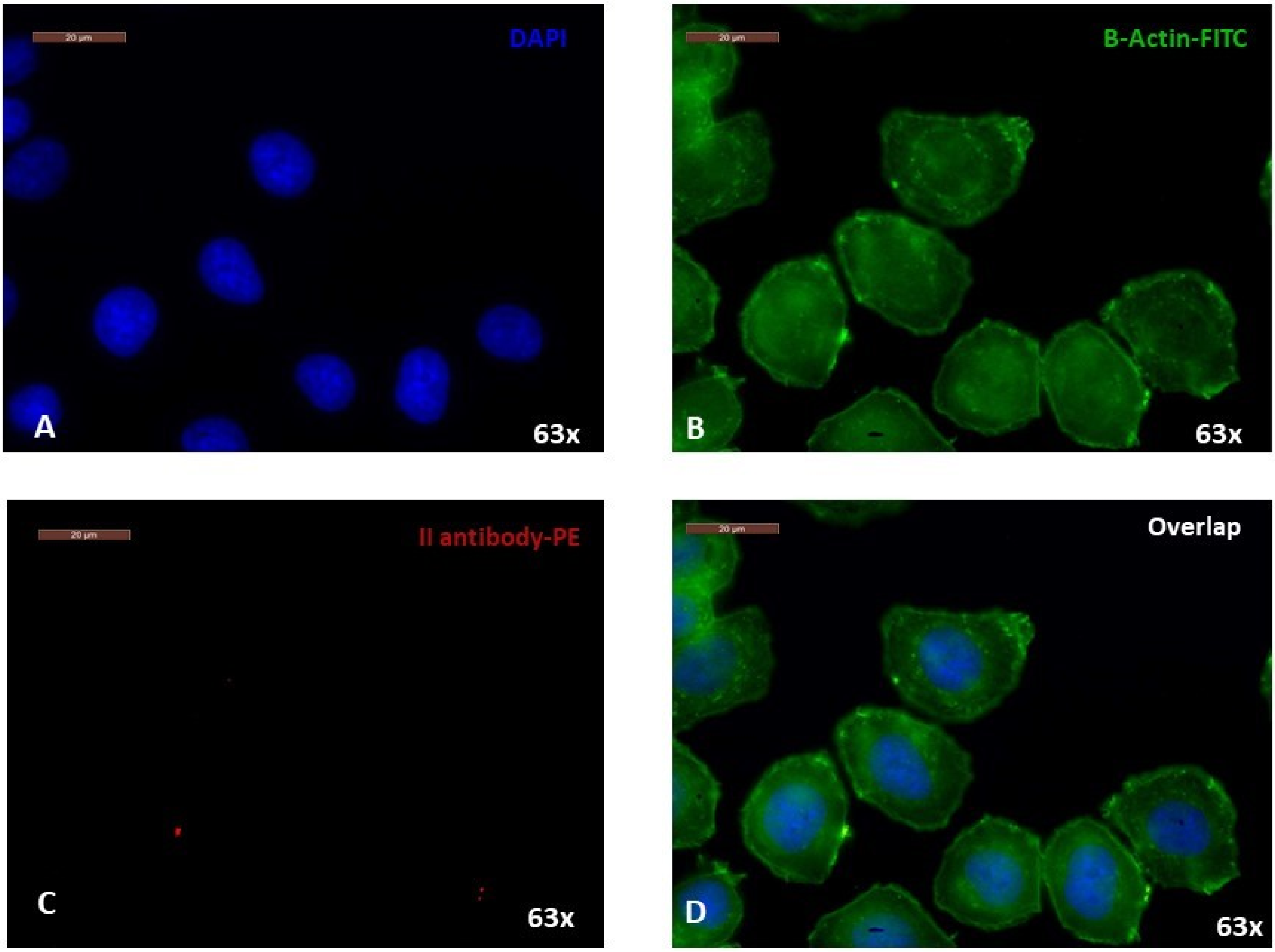

Supplementary Figure S7. Immunofluorescence assay on SKBR3 cells. A) Nuclei staining with DAPI (blue) B) β-actin staining with FITC-conjugated secondary antibody. C) PE-conjugated II antibody. D) Overlapping of FITC, PE, and DAPI channels. Magnification 63 × . Scale bars 20 µm.

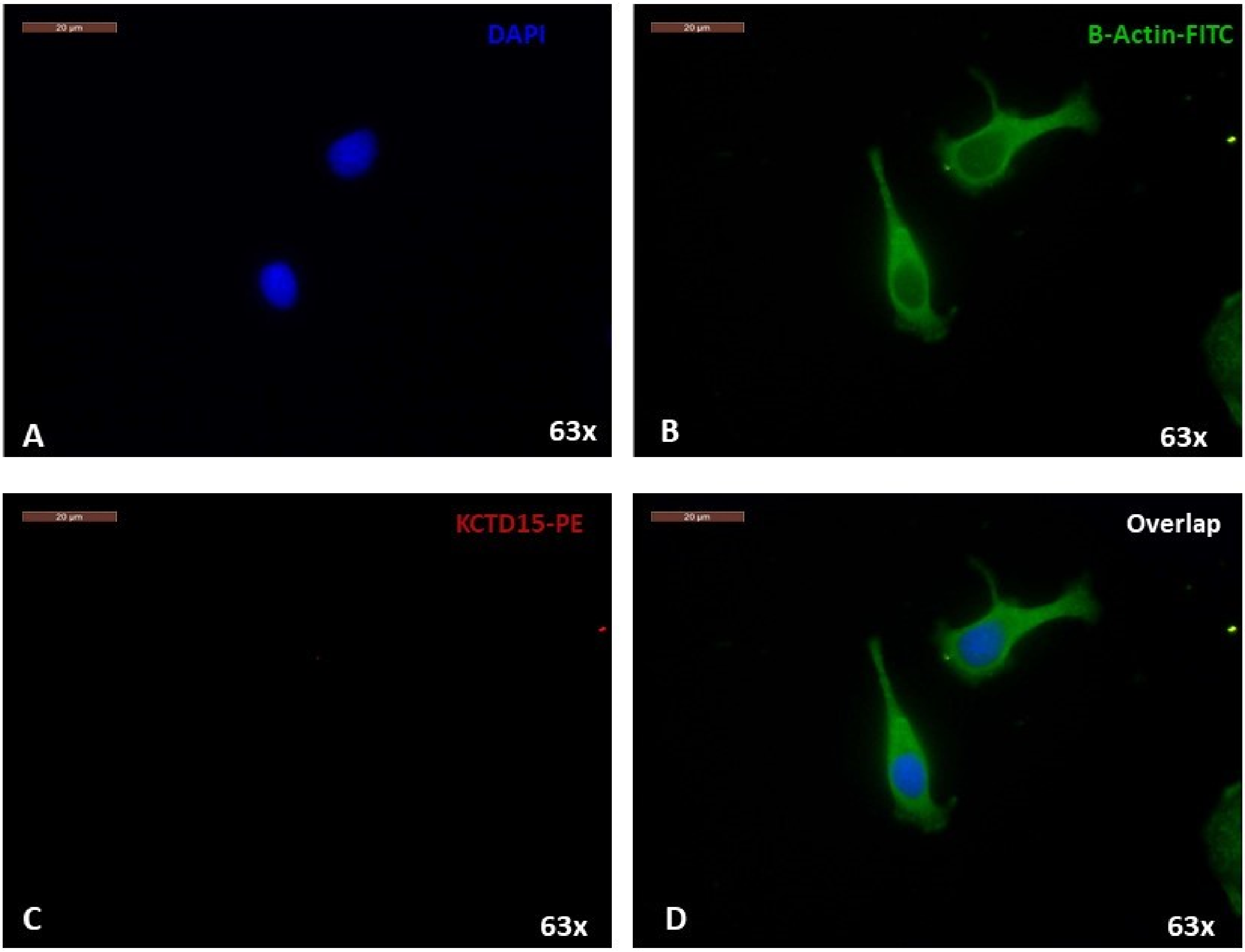

Supplementary Figure S8. Immunofluorescence assay on MDA-MB-231 cells. A) Nuclei staining with DAPI (blue) B) β-actin staining with FITC-conjugated secondary antibody. C) Endogenous KCTD15 labeled with PEconjugated secondary antibody. D) Overlapping of FITC, PE, and DAPI channels. Magnification 63 × . Scale bars 20 µm

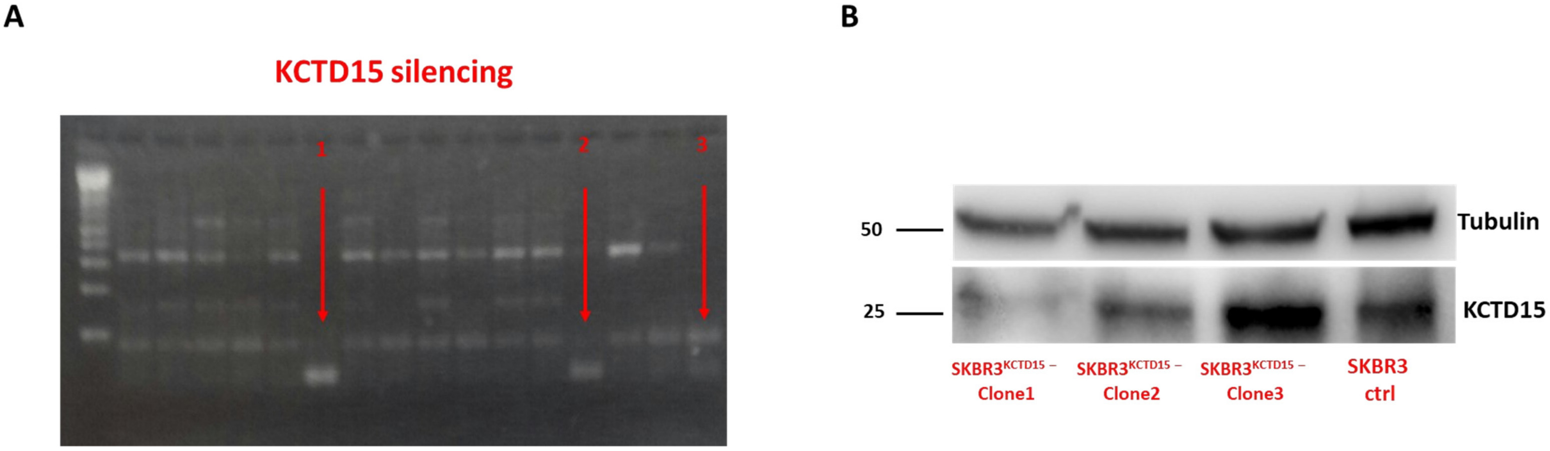

Supplementary Figure S9. CRISPR/CAS9 KCTD15 silencing selection clones. A) Agarose gel of DNA derived from SKBR3 KCTD15 silenced clones. In the first lane the DNA markers are reported whereas in the second the PCR amplified DNA of the exon 1 derived from SKBR3^{ctrl} is shown. The other lanes report the PCR amplified DNA of the exon 1 derived from the putative KCTD15-silenced clones. The red arrows denotes the three clones (clone1 to clone3) with reduced/absent DNA of the exon 1. B) WB shows KCTD15 expression of these three clones. Only in the case of clone1 KCTD15 was undetactable in the WB. Numbers represent the molecular weight of the protein marker expressed in kDa.

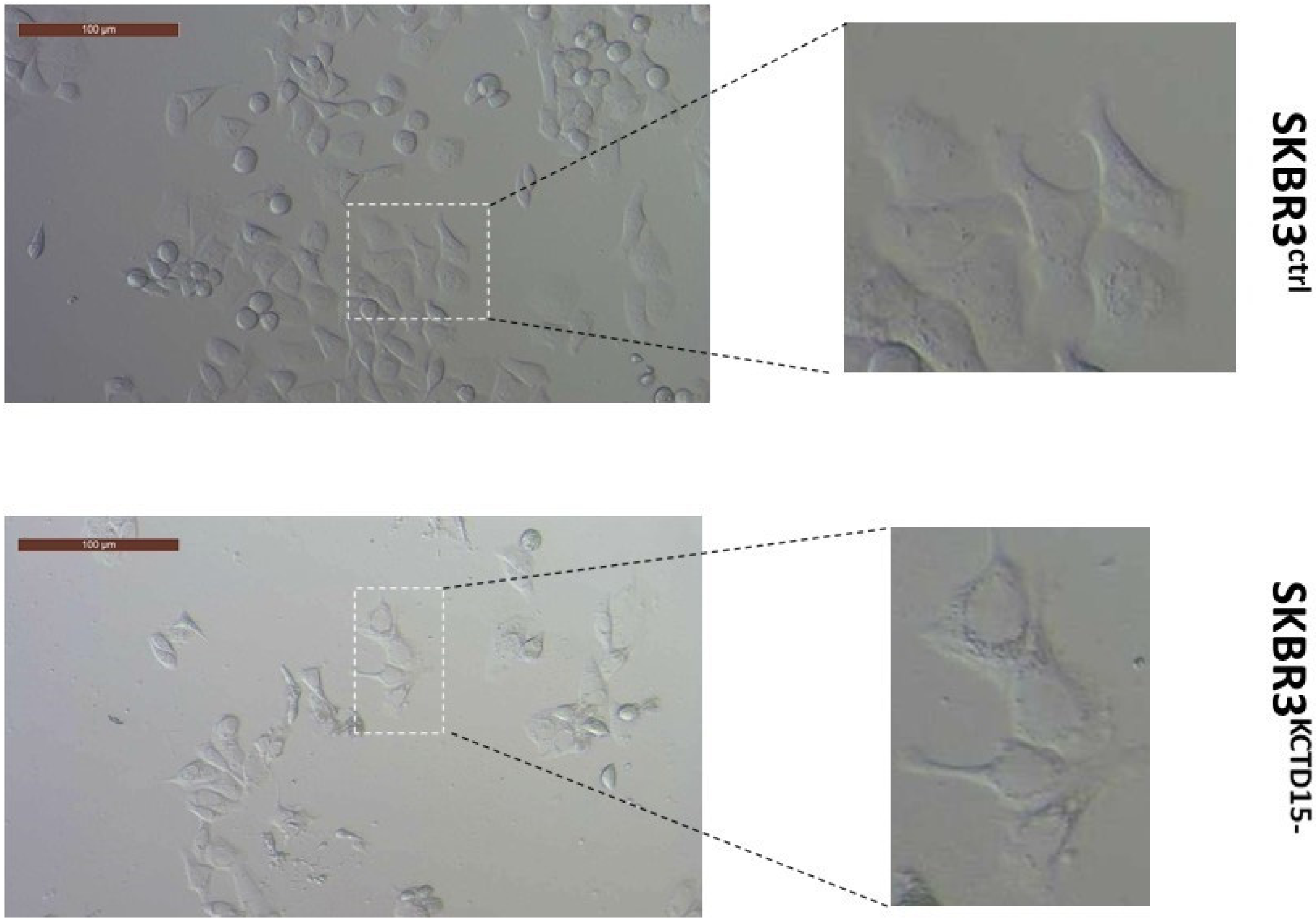

Supplementary Figure S10. Morphological evaluation of SKBR3$^{KCTD15-}$ cells. Morphological analyses of SKBR3^{ctrl} (upper panels) and SKBR3$^{KCTD15-}$ (lower panel) selected clone by brightfield microscopy. In the insets a zommed section. Magnification 20 × . Scale bars 100 µm.

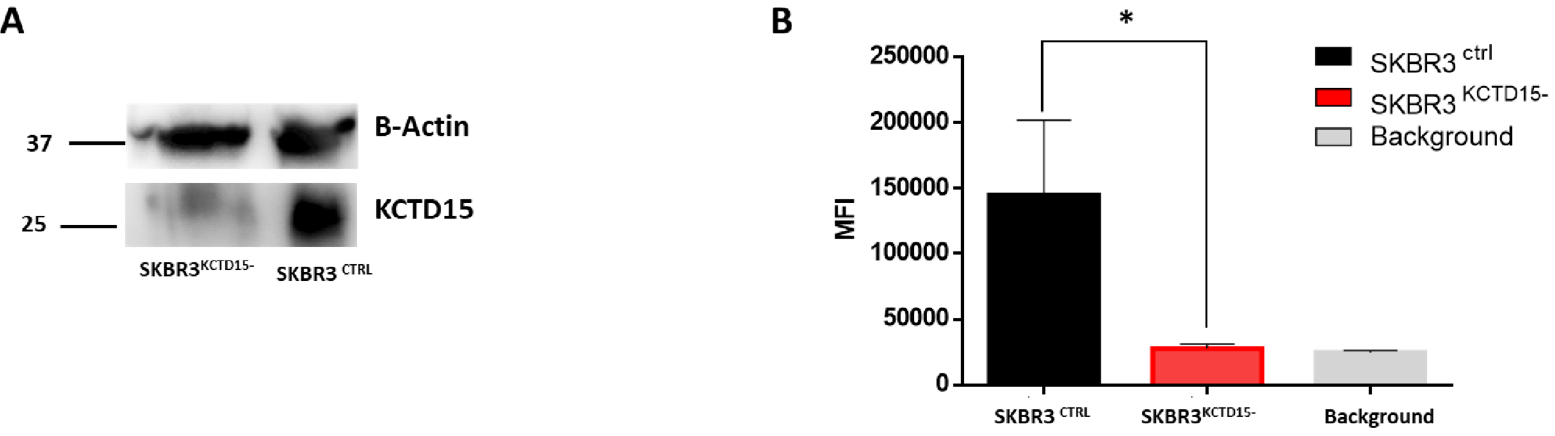

Supplementary Figure S11. **CRISP/CAS9 KCTD15 silencing in SKBR3**. A) WB shows KCTD15 expression in SKBR3$^{KCTD15-}$ compared with SKBR3 control after 72h of cell culture. Numbers represent the molecular weight of the protein marker expressed in kDa. B) Bar-plots display fluorescence intensity (in terms of the median of three independent experiments and SD) of KCTD15 in SKBR3 control (Black) and SKBR3$^{KCTD15-}$ (red) after 48h of culture. The light gray plot represents the fluorescence background. *<0,05, Unpaired Ttest.

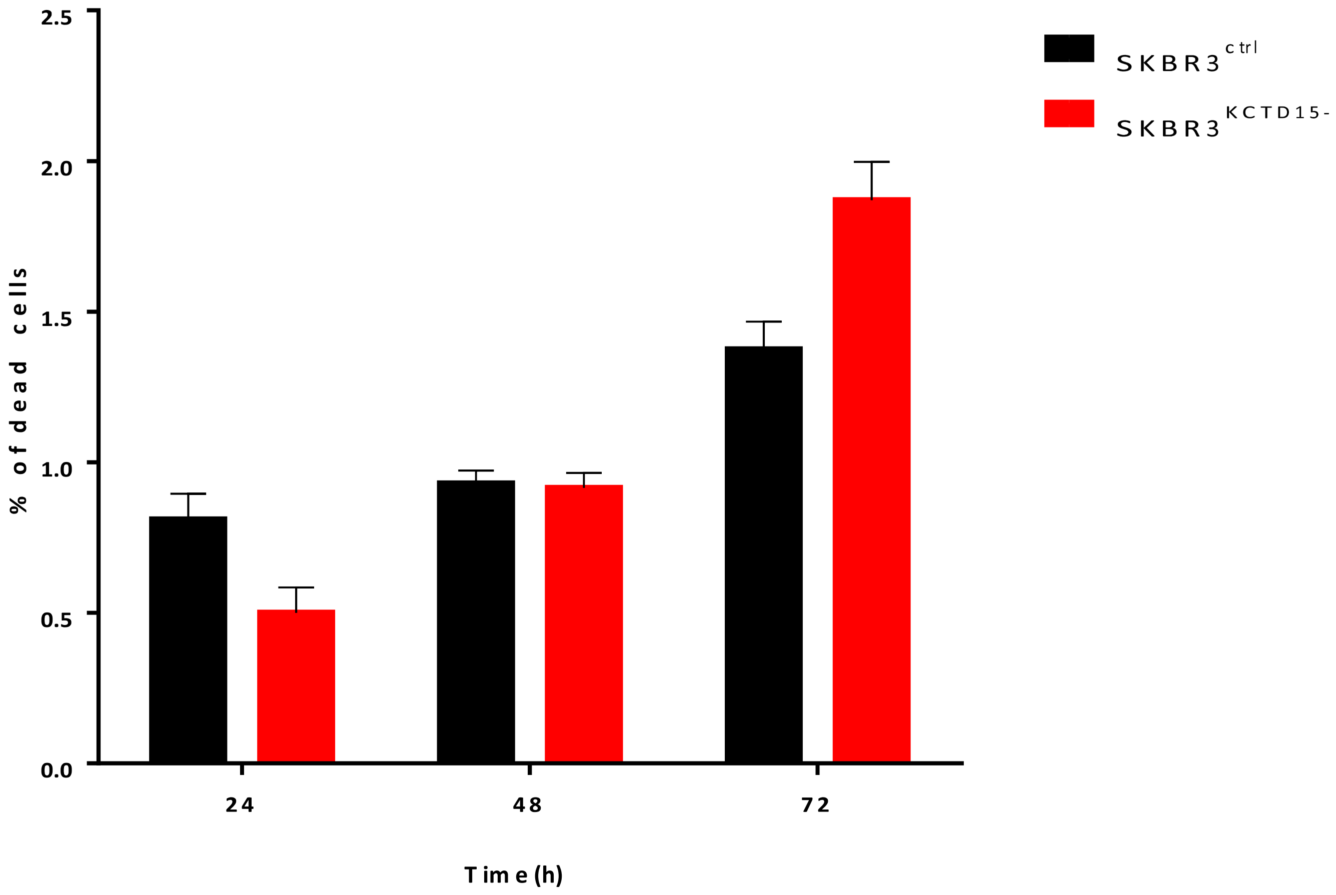

Supplementary Figure S12. **KCTD15 silencing not altered SKBR3 viability**. Bar-plots display percentage of SKBR3^{ctrl}(black bar) and SKBR3$^{KCTD15-}$ (red bar) positive at Propidium Iodide detected by cytofluorimetric analisys. Differences are not significative in every time points. Error bars represent the SD of three independent experiments.

A

T0

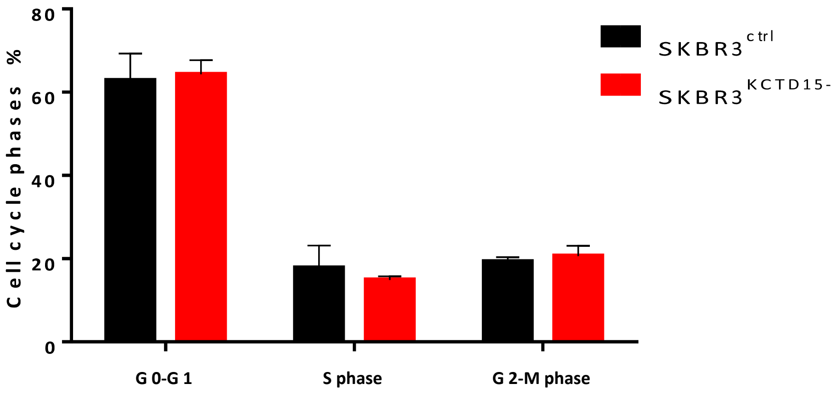

B

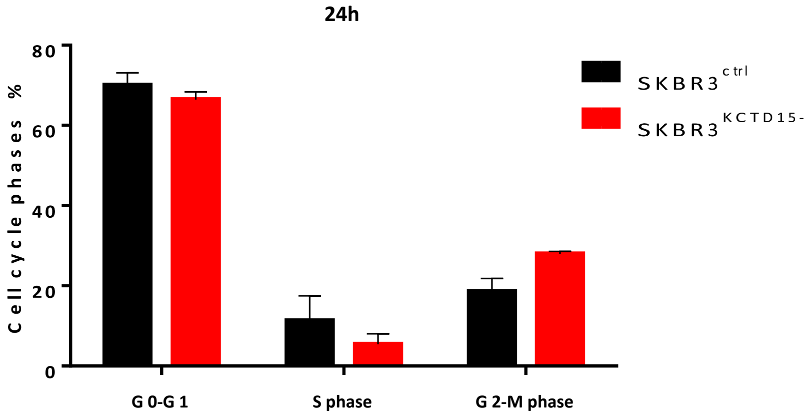

C

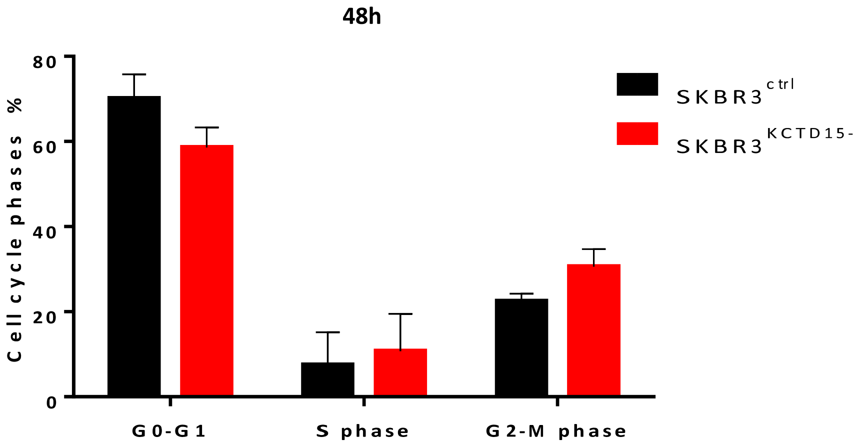

Supplementary Figure S13. Histogram representation of cell cycle phase percentages, at T0 (A), 24h (B) and 48h (C) of active growth of SKBR3^{ctrl} (black bars) and SKBR3$^{KCTD15-}$ (red bars). Percentages derive from three independent experiments. Error bars indicate standard deviation.
